# Supplementary material for: The role of amygdala GABA neurons in controlling stress and reproduction in female mice
Source: Nat Commun. 2026 Mar 10;17:5690. doi: 10.1038/s41467-026-70364-9 (PMC13319269; doi:10.1038/s41467-026-70364-9)
Supplement: Supplementary file 2 — Reporting Summary [file 41467_2026_70364_MOESM2_ESM.pdf]

nature portfolio

Corresponding author(s): Krasimira Tsaneva-Atanasova
Kevin O'Byrne
Margaritis Voliotis

Last updated by author(s): 2026/02/13

Reporting Summary

Nature Portfolio wishes to improve the reproducibility of the work that we publish. This form provides structure for consistency and transparency in reporting. For further information on Nature Portfolio policies, see our Editorial Policies and the Editorial Policy Checklist.

Statistics

For all statistical analyses, confirm that the following items are present in the figure legend, table legend, main text, or Methods section.

n/a	Confirmed	
☐	☒	The exact sample size (*n*) for each experimental group/condition, given as a discrete number and unit of measurement
☐	☒	A statement on whether measurements were taken from distinct samples or whether the same sample was measured repeatedly
☐	☒	The statistical test(s) used AND whether they are one- or two-sided<br>*Only common tests should be described solely by name; describe more complex techniques in the Methods section.*
☒	☐	A description of all covariates tested
☐	☒	A description of any assumptions or corrections, such as tests of normality and adjustment for multiple comparisons
☐	☒	A full description of the statistical parameters including central tendency (e.g. means) or other basic estimates (e.g. regression coefficient) AND variation (e.g. standard deviation) or associated estimates of uncertainty (e.g. confidence intervals)
☐	☒	For null hypothesis testing, the test statistic (e.g. *F*, *t*, *r*) with confidence intervals, effect sizes, degrees of freedom and *P* value noted<br>*Give P values as exact values whenever suitable.*
☒	☐	For Bayesian analysis, information on the choice of priors and Markov chain Monte Carlo settings
☐	☒	For hierarchical and complex designs, identification of the appropriate level for tests and full reporting of outcomes
☐	☒	Estimates of effect sizes (e.g. Cohen's *d*, Pearson's *r*), indicating how they were calculated

Our web collection on statistics for biologists contains articles on many of the points above.

Software and code

Policy information about availability of computer code

Data collection	The Data Acquisition Software (IDAS; Inscopix, Inc.) was used to perform calcium imaging. The video data was processed with the Inscopix Data Processing Software (IDPS). For the recordings with GCaMP signals from GABAergic neurons we used the MATLAB (version R2022a) implementation of the open-source algorithm NoRMCorre to perform motion correction.
Data analysis	Data analysis utilized Python v 3.12 with the following packages: scipy v1.15., sklearn v1.6.1., statsmodels 0.14.4. The code to reproduce data analysis is at DOI: 10.24378/exe.31018072.

For manuscripts utilizing custom algorithms or software that are central to the research but not yet described in published literature, software must be made available to editors and reviewers. We strongly encourage code deposition in a community repository (e.g. GitHub). See the Nature Portfolio guidelines for submitting code & software for further information.

Data

Policy information about availability of data

All manuscripts must include a data availability statement. This statement should provide the following information, where applicable:

- Accession codes, unique identifiers, or web links for publicly available datasets
- A description of any restrictions on data availability
- For clinical datasets or third party data, please ensure that the statement adheres to our policy

The main data supporting the results in this study are available within the paper and its Supplementary Information. The data sets generated during this study are publicly available at Figshare (DOI:10.24378/exe.31021447). Source data are provided with this paper for reproducing all Figures in the manuscript and its

Supplementary Information.

Research involving human participants, their data, or biological material

Policy information about studies with human participants or human data. See also policy information about sex, gender (identity/presentation), and sexual orientation and race, ethnicity and racism.

Reporting on sex and gender	N/A
Reporting on race, ethnicity, or other socially relevant groupings	N/A
Population characteristics	N/A
Recruitment	N/A
Ethics oversight	N/A

Note that full information on the approval of the study protocol must also be provided in the manuscript.

Field-specific reporting

Please select the one below that is the best fit for your research. If you are not sure, read the appropriate sections before making your selection.

[x] Life sciences   [ ] Behavioural & social sciences   [ ] Ecological, evolutionary & environmental sciences

For a reference copy of the document with all sections, see nature.com/documents/nr-reporting-summary-flat.pdf

Life sciences study design

All studies must disclose on these points even when the disclosure is negative.

Sample size	According to previous data on LH pulse dynamics in rodents from our laboratory and publications, we performed power calculations assuming a minimum detectable difference in mean with expected standard deviation of 20%. Using alpha 0.05 and power 0.9, as well as considering the number of groups compared, we calculated the sample size. The average LH inter-pulse interval was calculated for the control period and the stimulation period in both experimental and control mice. Statistical significance was assessed using a 2-way ANOVA test.
Data exclusions	The data analysis was performed in a blinded manner to minimise bias. The data of the animals were excluded from the final analysis only if post-hoc histological verification revealed (1) incorrect anatomical targeting of viral vectors to the MePD or (2) misplaced optical fibers or GRIN lenses. In total, 6 mice were excluded based on these criteria. All other data were included in the statistical analysis.
Replication	Continuous serial blood samples (every 5 min) were collected for LH pulse data instead of mean or individual samples. It is well established and well accepted method in our field.
Randomization	The experiments were conducted using a randomized design. The mice of the same genotype and age were randomly assigned to either the experimental group (receiving the active viral vector) or the control group (receiving a control virus). This randomization was performed within each animal cage to ensure that environmental factors were balanced across groups. Animals were randomly assigned to treatment and control groups using a random draw to minimize selection bias.
Blinding	The investigators were not blinded to allocation during experiments or outcome assessment.

Reporting for specific materials, systems and methods

We require information from authors about some types of materials, experimental systems and methods used in many studies. Here, indicate whether each material, system or method listed is relevant to your study. If you are not sure if a list item applies to your research, read the appropriate section before selecting a response.

Materials & experimental systems

n/a	Involved in the study	
[ ]	[x]	Antibodies
[x]	[ ]	Eukaryotic cell lines
[x]	[ ]	Palaeontology and archaeology
[ ]	[x]	Animals and other organisms
[x]	[ ]	Clinical data
[x]	[ ]	Dual use research of concern
[x]	[ ]	Plants

Methods

n/a	Involved in the study	
[x]	[ ]	ChIP-seq
[x]	[ ]	Flow cytometry
[x]	[ ]	MRI-based neuroimaging

Antibodies

Antibodies used	The following antibodies were used in the assay: (i) coating antibody (RRID: AB_2665514, monoclonal antibovine LH beta subunit antiserum, 518B7, University of California, CA, USA; Lot No. 13; dilution 1:10,000); (ii) anti-LH antibody (polyclonal rabbit LH antiserum, AFP240580Rb, RRID: AB_2665533; National Hormone & Peptide Program, CA, USA; dilution 1:40) (iii) HRP-linked donkey anti-rabbit IgG, (Cat. No. NA934, RRID: AB_772206, VWR International/GE Healthcare, UK)
Validation	Validation for LH ELISA: Steyn FJ, Wan Y, Clarkson J, Veldhuis JD, Herbison AE, Chen C. Development of a methodology for and assessment of pulsatile luteinizing hormone secretion in juvenile and adult male mice. Endocrinology. 2013;154(12):4939-4945.

Animals and other research organisms

Policy information about studies involving animals; ARRIVE guidelines recommended for reporting animal research, and Sex and Gender in Research

Laboratory animals	VGAT-Cre-tdTomato mice were generated by crossbreeding Vgat-cre homozygous mice (Jax stock #028862, B6J.129S6(FVB)-Slc32a1tm2(cre)lowl/MwarJ; Jackson Laboratory, Bar Harbor, ME, USA) with adult homozygous tdTomato mice (Jax stock #007909, B6.Cg-Gt(ROSA)26Sortm9(CAGtdTomato)Hze/J, Jackson Laboratory). VGAT-Cre-tdTomato mice were genotyped using PCR to determine heterozygosity for VGAT-Cre (primers 5′-3′: common, 12785—CTTCGTCATCGGCGGCATCTG, Sigma-Aldrich; wild-type reverse 12786— CAGGGCGATGTGGAATAGAAA, Sigma-Aldrich; mutant reverse oIMR8292—CCAAAAGACGGCAATATGGT, Sigma-Aldrich) and tdtomato (primers 5′-3′: wild-type forward oIMR9020—AAGGGAGCTGCAGTGGAGTA, Sigma-Aldrich; wild-type reverse oIMR9021—CCGAAAATCTGTGGGAAGTC, Sigma-Aldrich; mutant reverse WPRE oIMR9103— GGCATTAAAGCAGCGTATCC, Sigma-Aldrich; mutant forward tdTomato oIMR9105—CTGTTCCTGTACGGCATGG, Sigma-Aldrich).<br>UCN3-Cre heterozygous mice (strain Tg(Ucn3-cre)KF43Gsat/ Mmucd, MMRRC GENSAT) were crossbred with VGAT-Flpo heterozygous mice (Jax stock #007909,B6.Cg-Slc32a1 2A-Flpo-D:Het) to obtain female double heterozygous UCN3-Cre::VGATFlpo mice (Slc32a1 2A-Flpo-D:Het|Ucn3-Cre:Het|Rosa26<tomato>WT); genotyped using PCR for the detection of heterozygosity (primers 5′- 3′: UCN3 Forward: CGAAGTCCCTCTCACACCTGGTT, Sigma-Aldrich; Cre Reverse: CGGCAAACGGAC-AGAAGCATT, Sigma-Aldrich; Slc32a1 Mutant Forward: TGC ATC GCA TTG TCT GAG TAG, Sigma-Aldrich; Slc32a1 Mutant Reverse: GAC AGC CGT GAA CAG AAG G, Sigma-Aldrich).<br>Female mice, aged 8 to 10 weeks, weighing between 20 g and 25 g
Wild animals	N/A
Reporting on sex	This study focuses only on one sex - females, and only ovariectomized mice were used for controling the variables of the experiment.
Field-collected samples	N/A
Ethics oversight	All animal procedures performed were approved by the King's College London Animal Welfare and Ethical Review Body. Procedures were in accordance with UK Home Office regulation and under Project Licence Number PP4067293

Note that full information on the approval of the study protocol must also be provided in the manuscript.

Plants

Seed stocks	N/A
Novel plant genotypes	N/A
Authentication	N/A
