# Supplementary figures and images for: The role of amygdala GABA neurons in controlling stress and reproduction in female mice
Source: Nat Commun. 2026 Mar 10;17:5690. doi: 10.1038/s41467-026-70364-9 (PMC13319269; doi:10.1038/s41467-026-70364-9)

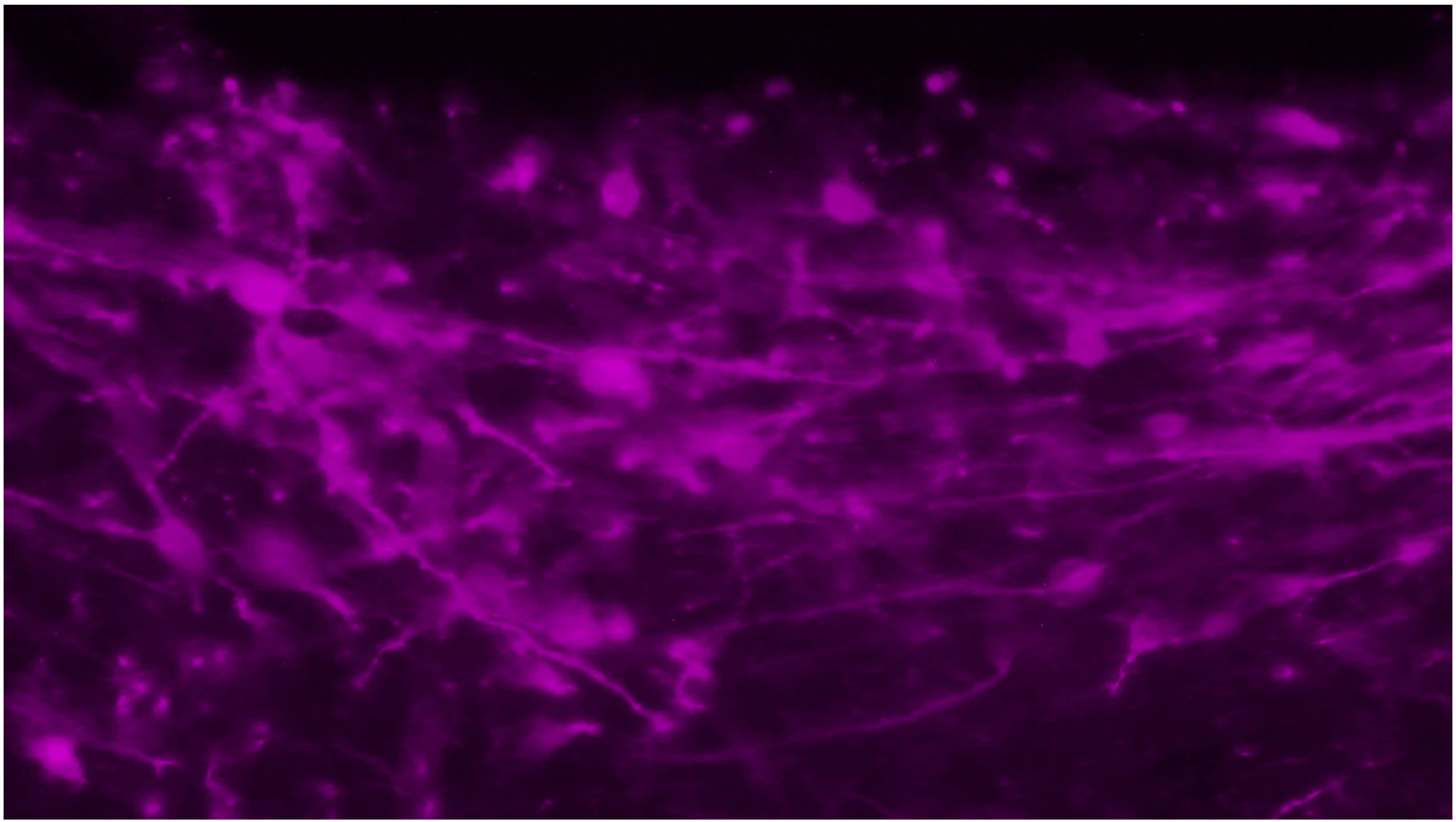

Supplement: Supplementary file 4 — Source data [file 41467_2026_70364_MOESM4_ESM.zip › SourceFiles/Fig 1E.tiff]

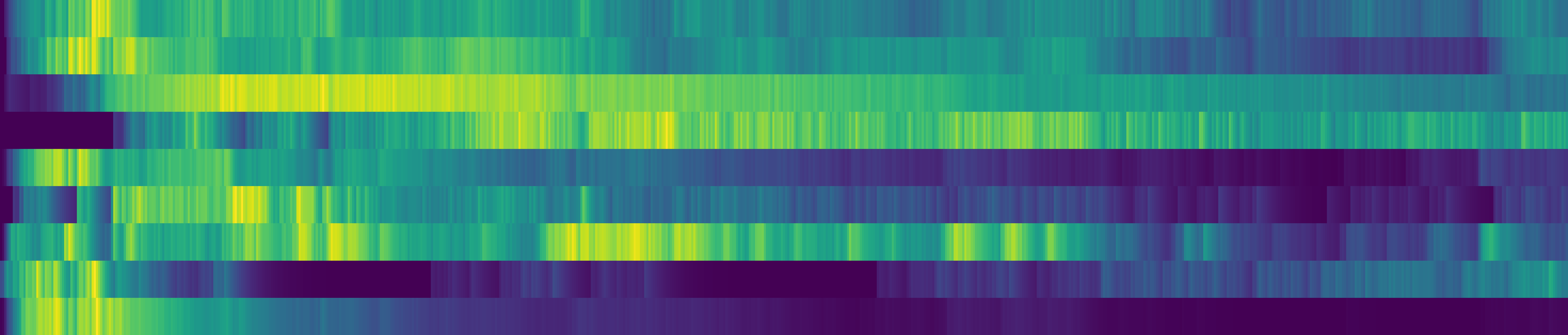

Supplement: Supplementary file 4 — Source data [file 41467_2026_70364_MOESM4_ESM.zip › SourceFiles/Figure3A.tiff]

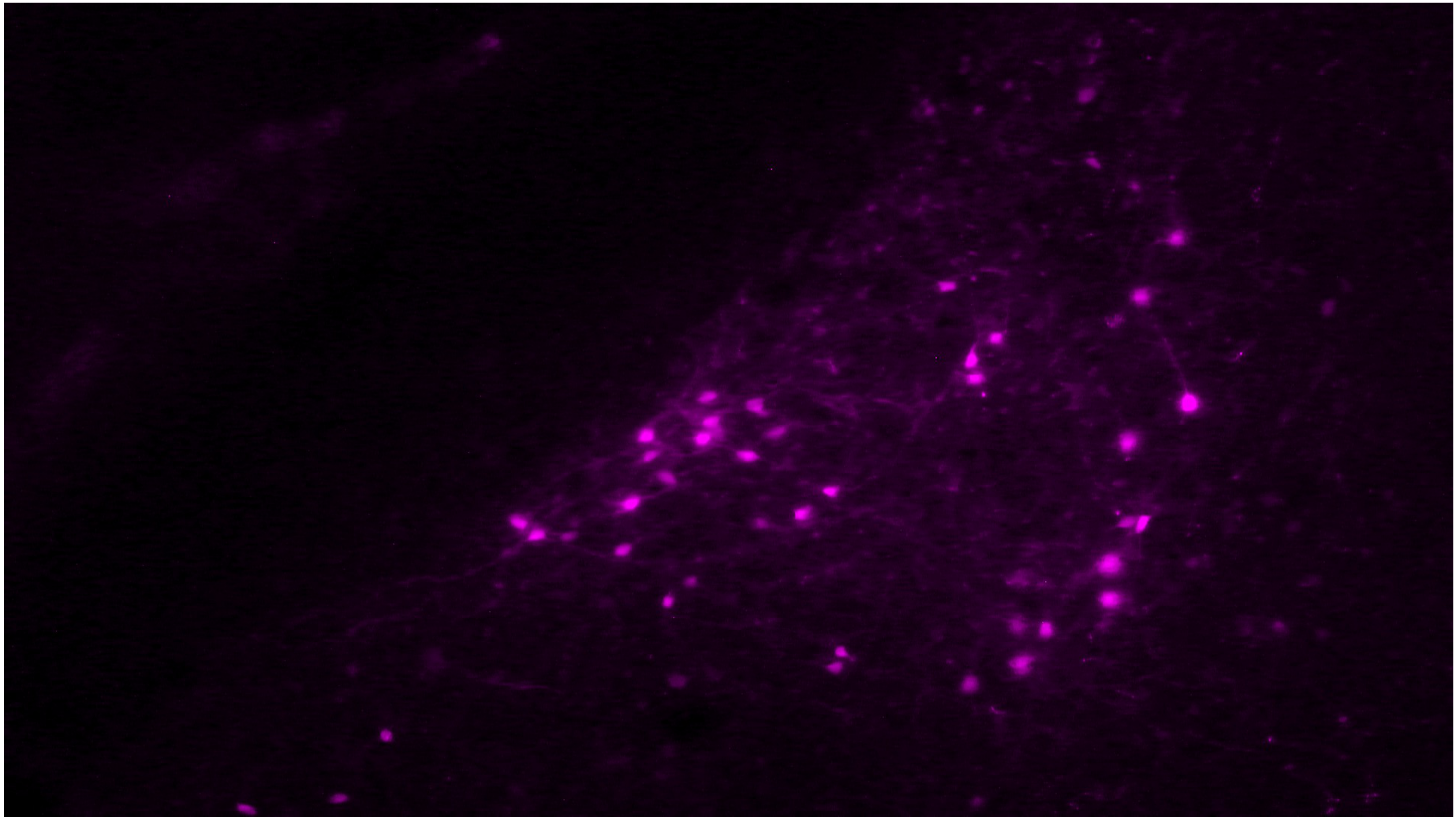

Supplement: Supplementary file 4 — Source data [file 41467_2026_70364_MOESM4_ESM.zip › SourceFiles/Fig 6N.tiff]

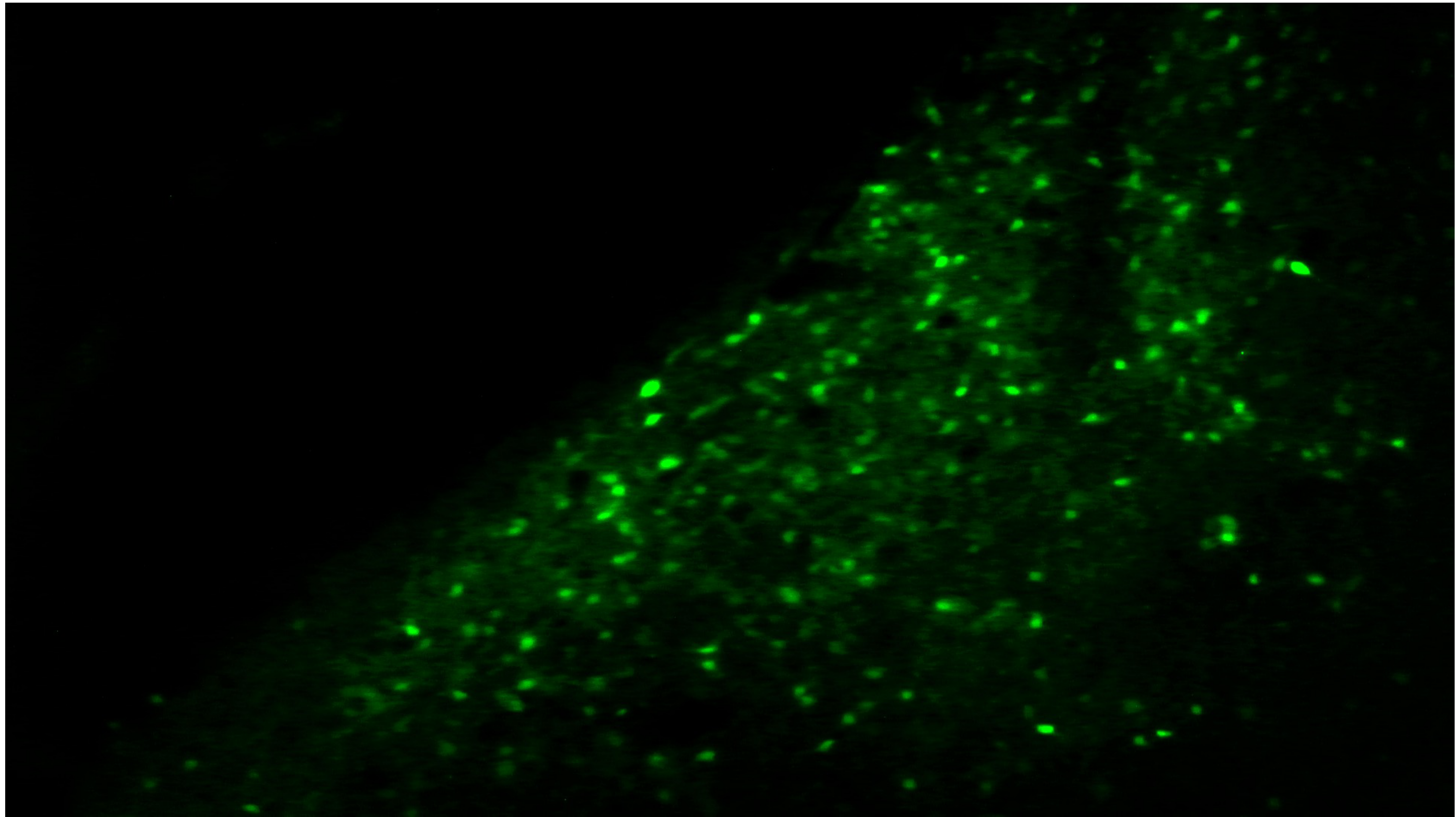

Supplement: Supplementary file 4 — Source data [file 41467_2026_70364_MOESM4_ESM.zip › SourceFiles/Fig 6O.tiff]

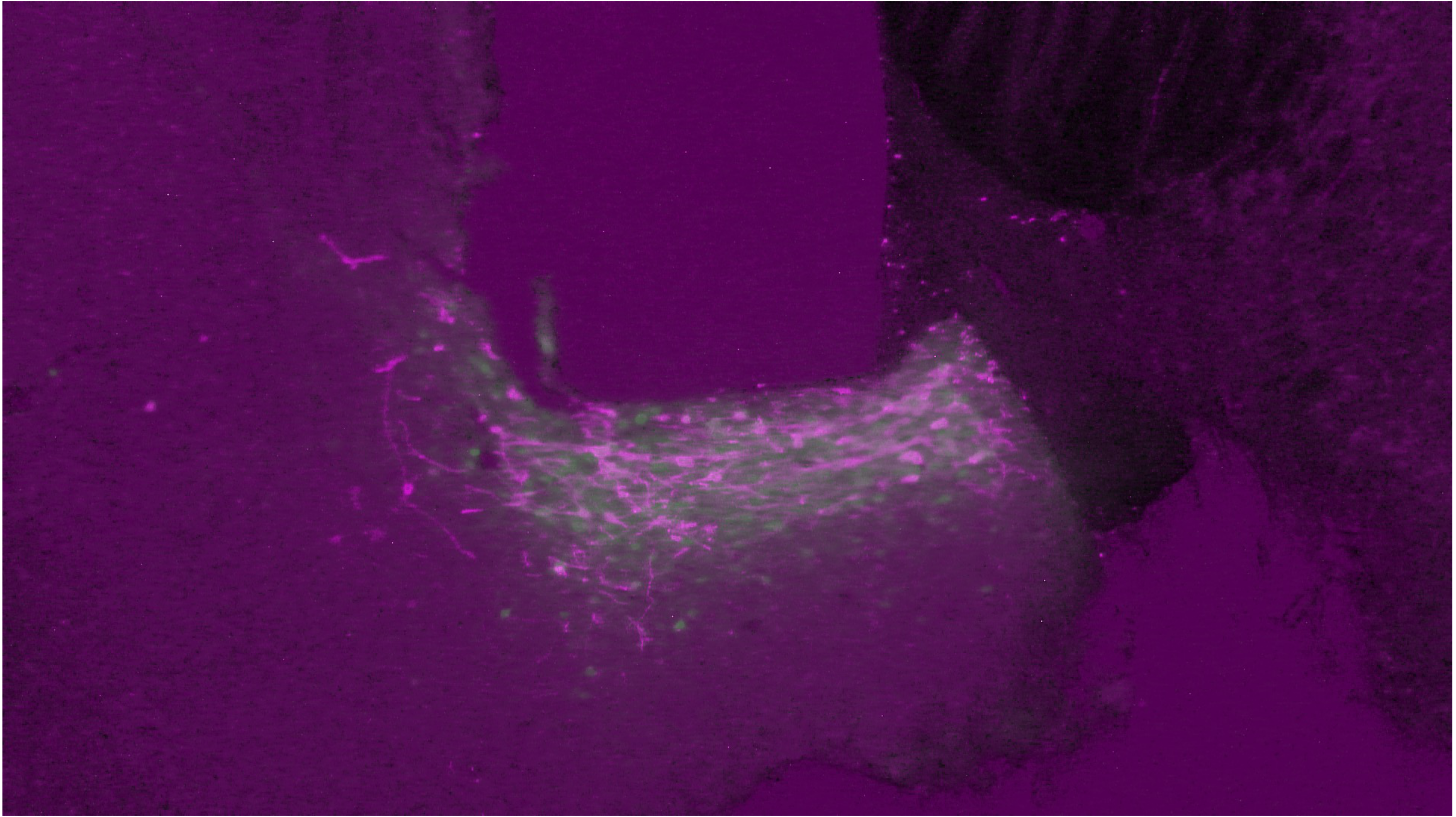

Supplement: Supplementary file 4 — Source data [file 41467_2026_70364_MOESM4_ESM.zip › SourceFiles/Fig 1D.tiff]

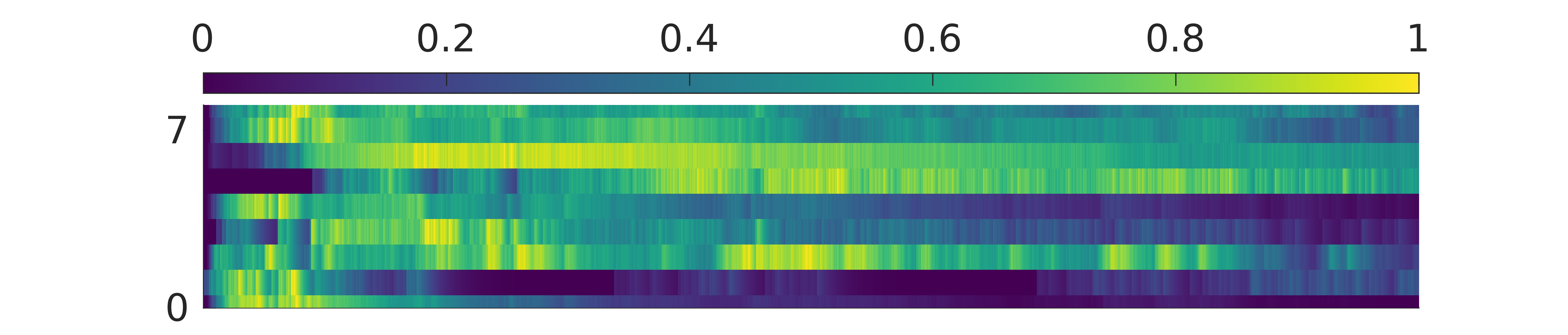

Supplement: Supplementary file 4 — Source data [file 41467_2026_70364_MOESM4_ESM.zip › SourceFiles/Fig3B.tiff]

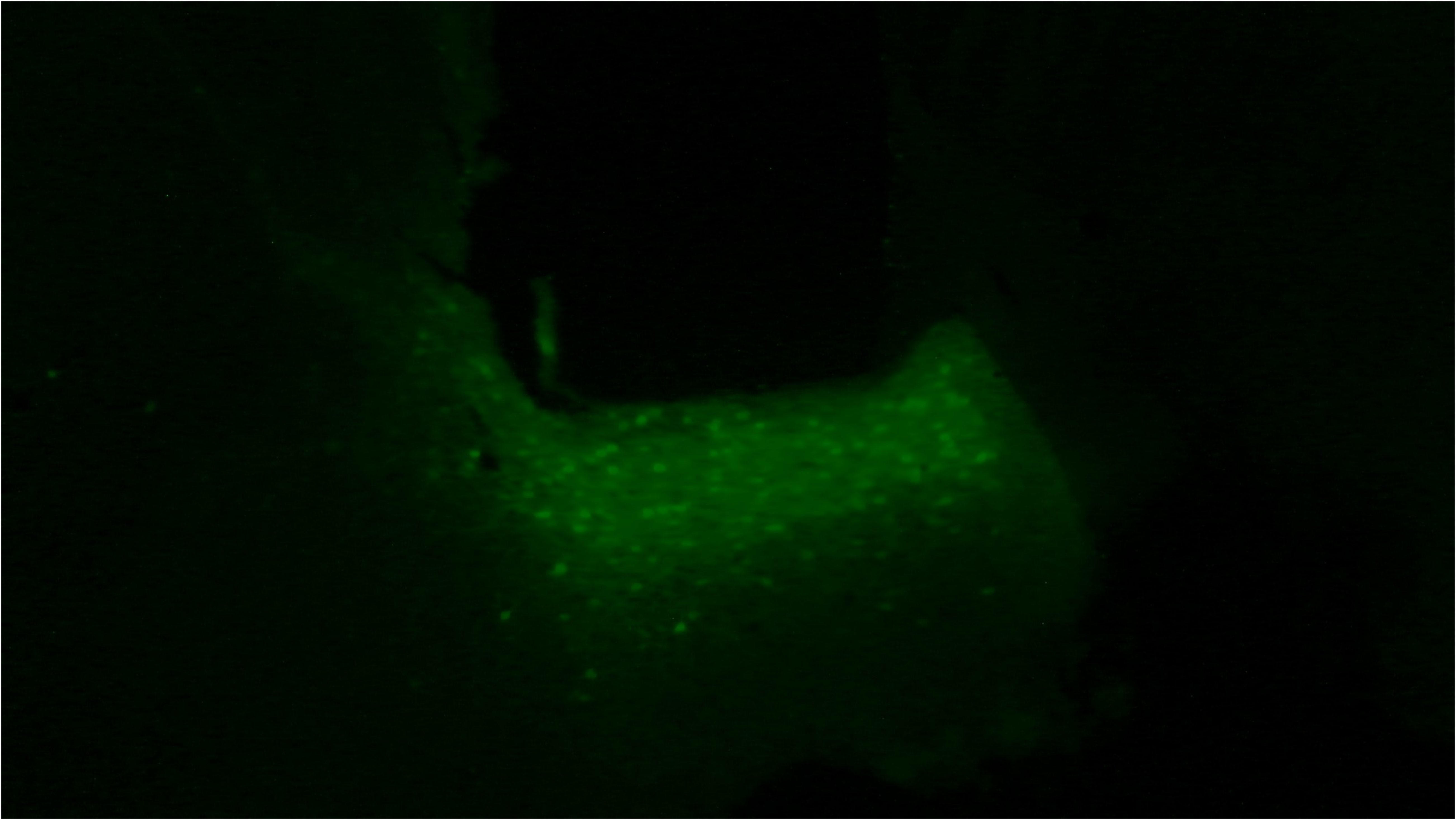

Supplement: Supplementary file 4 — Source data [file 41467_2026_70364_MOESM4_ESM.zip › SourceFiles/Fig 1C.tiff]

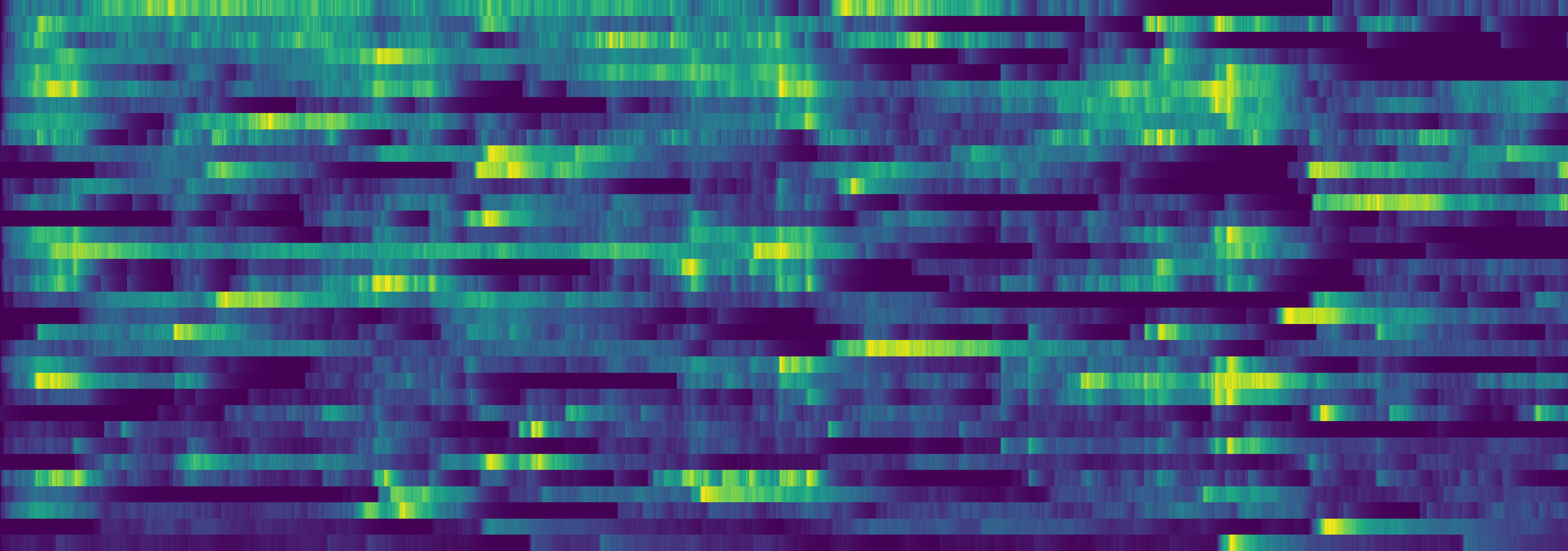

Supplement: Supplementary file 4 — Source data [file 41467_2026_70364_MOESM4_ESM.zip › SourceFiles/Figure1A(left).tiff]

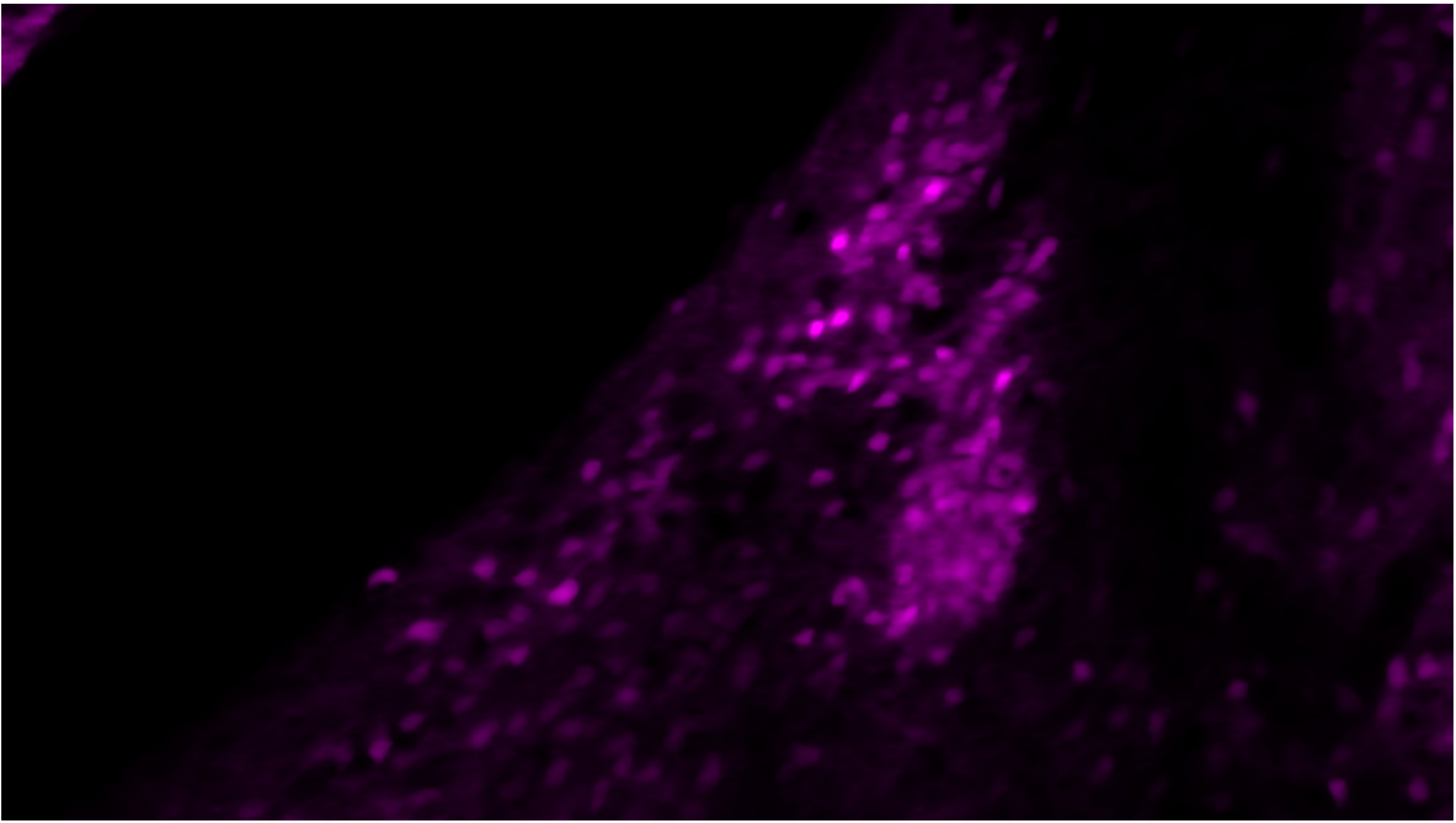

Supplement: Supplementary file 4 — Source data [file 41467_2026_70364_MOESM4_ESM.zip › SourceFiles/Fig 6E.tiff]

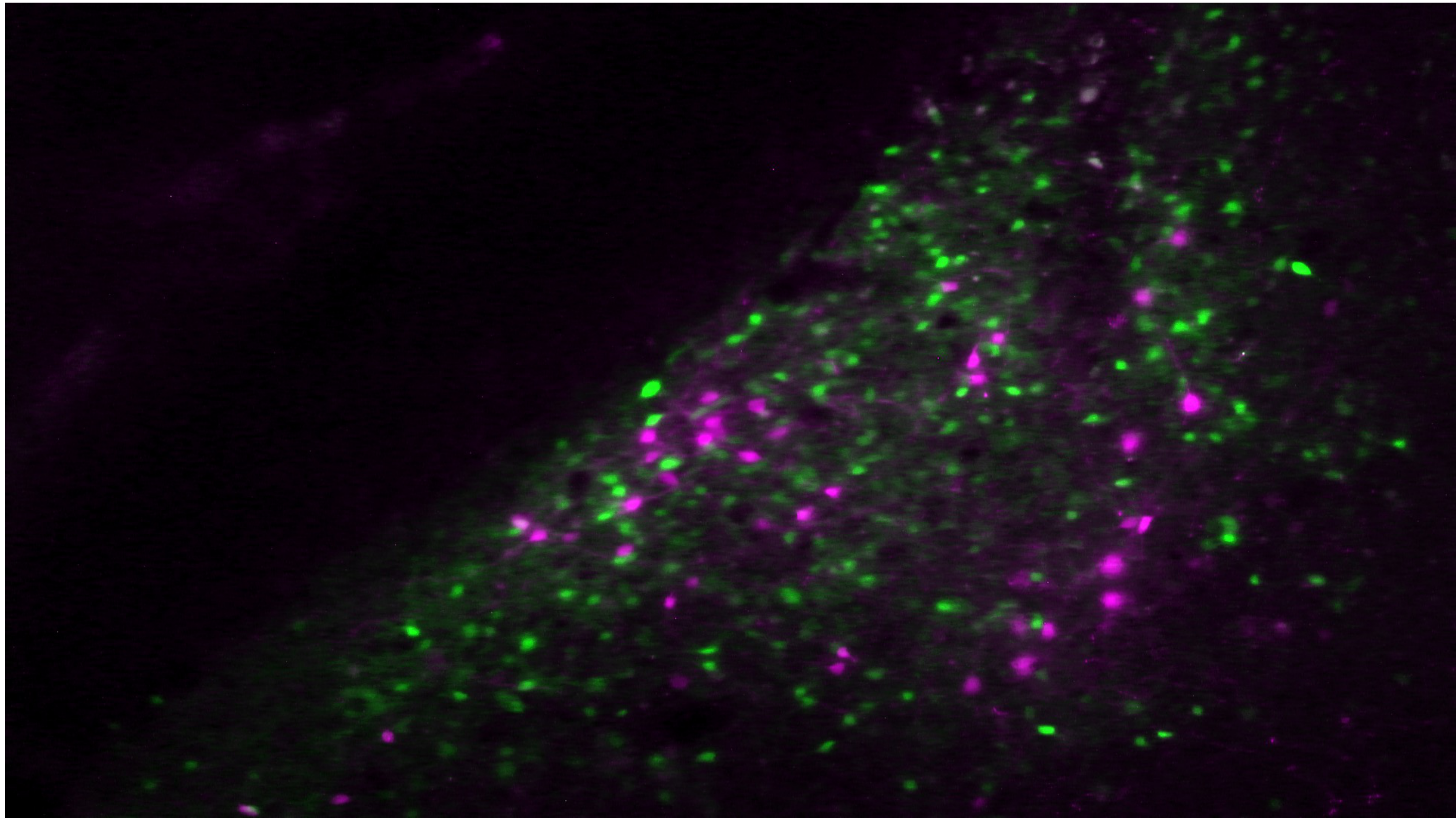

Supplement: Supplementary file 4 — Source data [file 41467_2026_70364_MOESM4_ESM.zip › SourceFiles/Fig 6P.tiff]

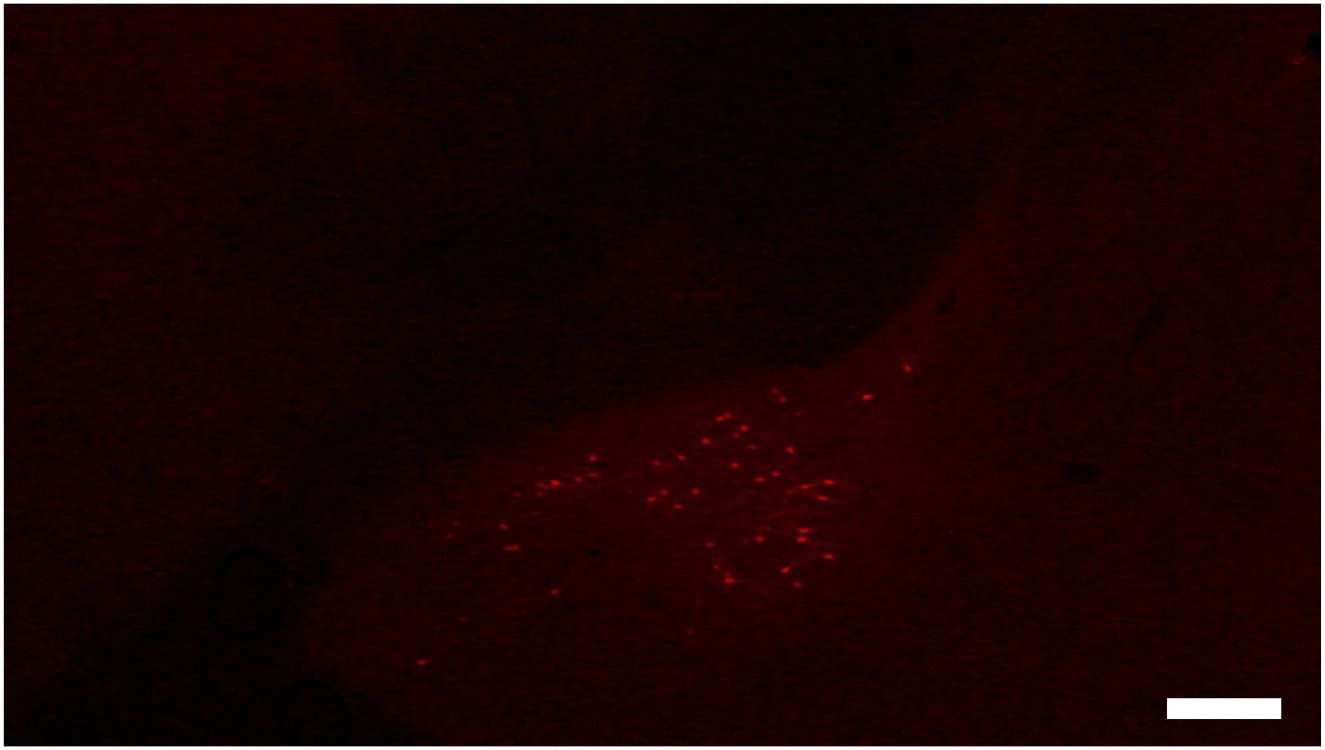

Supplement: Supplementary file 4 — Source data [file 41467_2026_70364_MOESM4_ESM.zip › SourceFiles/Fig5E.tiff]

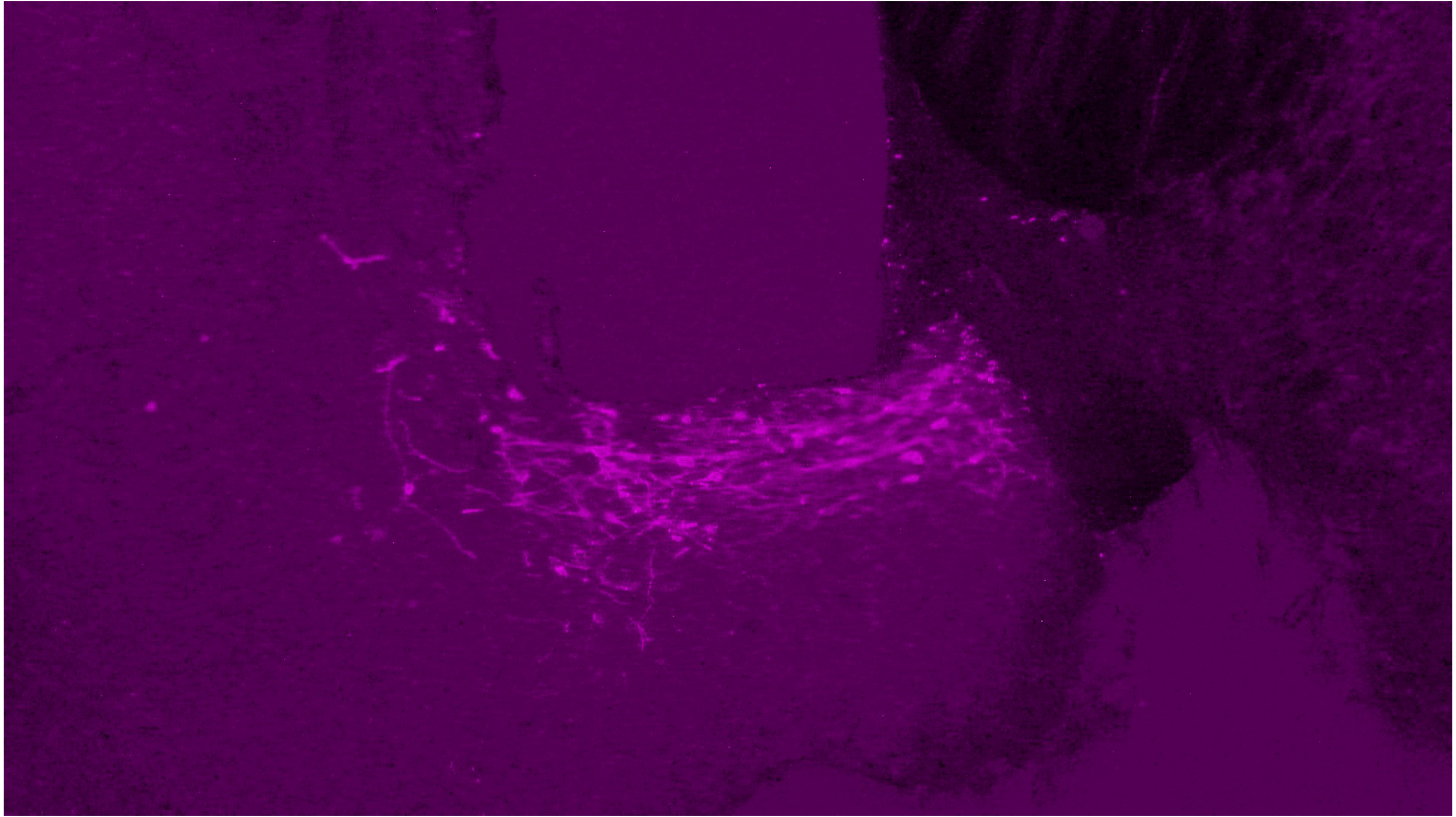

Supplement: Supplementary file 4 — Source data [file 41467_2026_70364_MOESM4_ESM.zip › SourceFiles/Fig 1B.tiff]

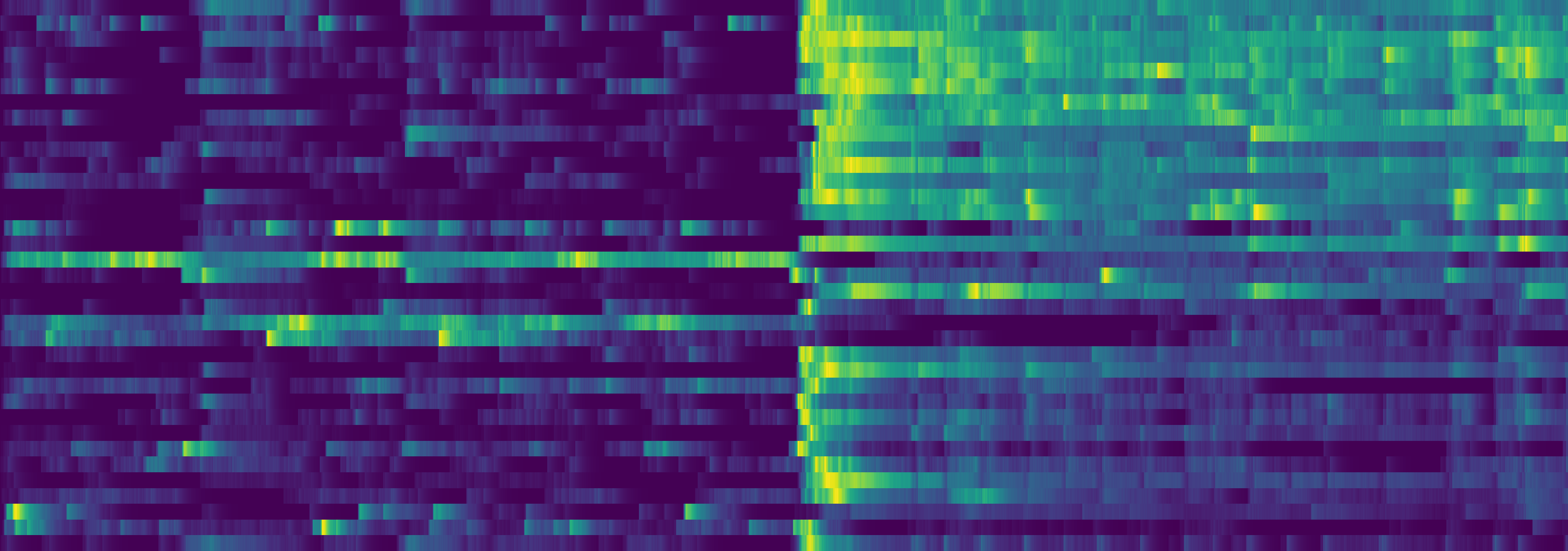

Supplement: Supplementary file 4 — Source data [file 41467_2026_70364_MOESM4_ESM.zip › SourceFiles/Figure1A(right).tiff]

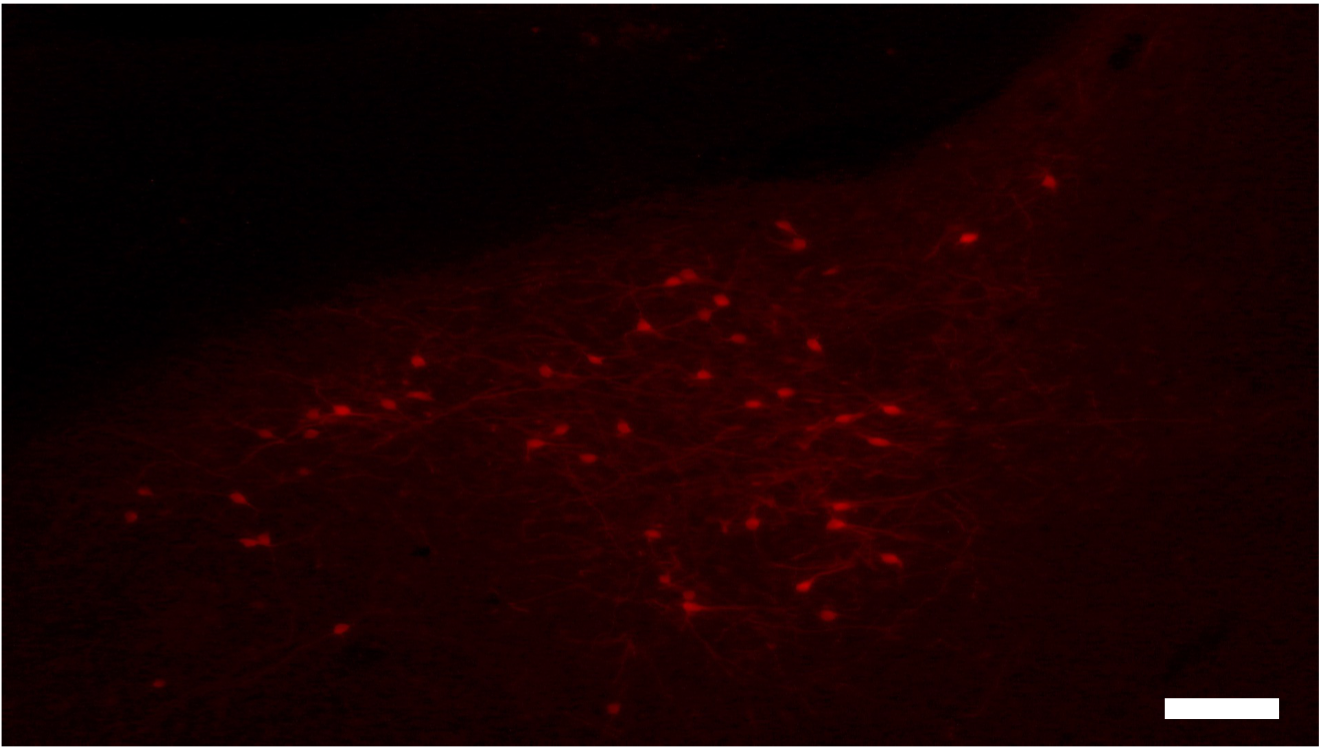

Supplement: Supplementary file 4 — Source data [file 41467_2026_70364_MOESM4_ESM.zip › SourceFiles/Fig5F.tiff]

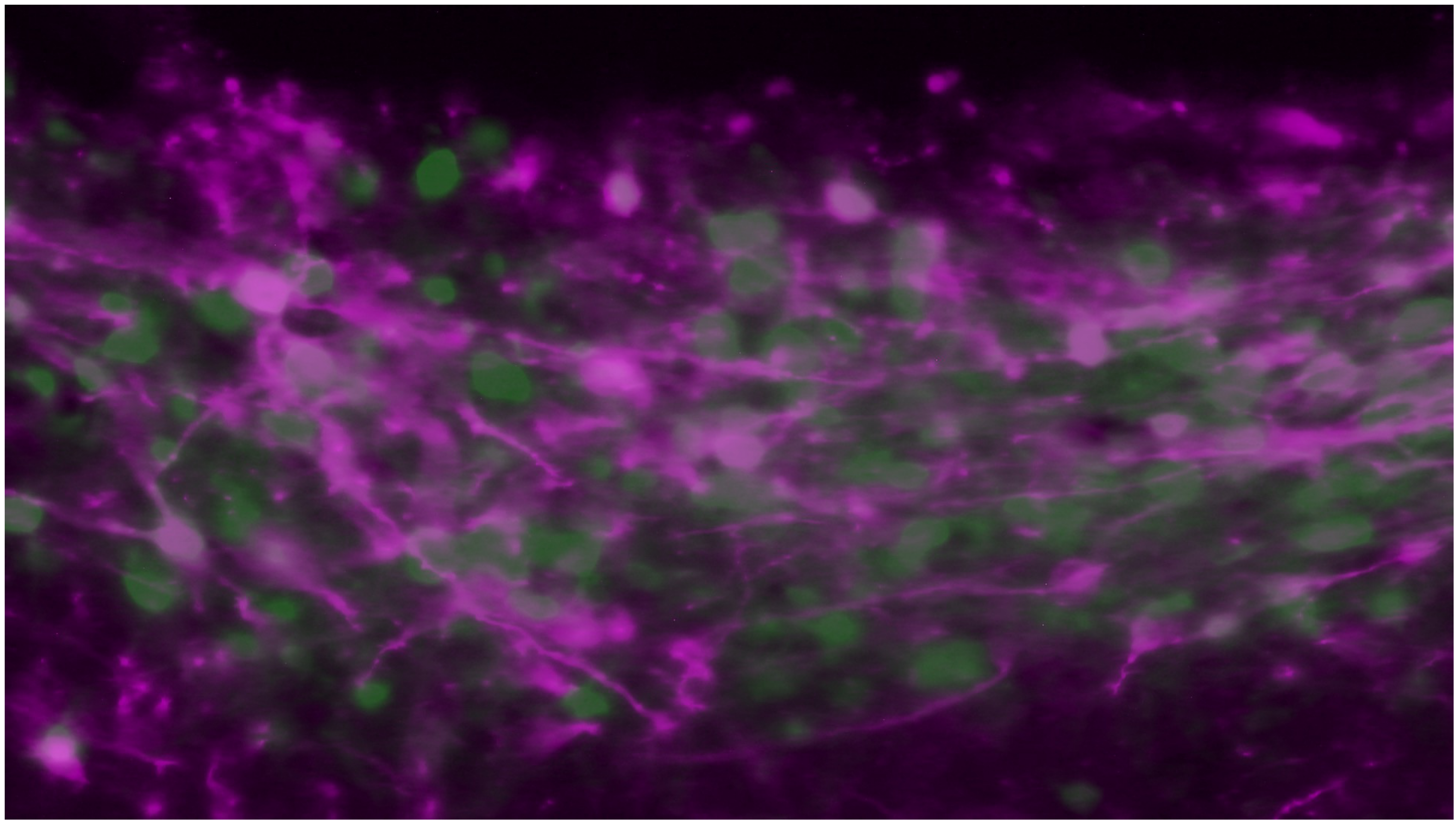

Supplement: Supplementary file 4 — Source data [file 41467_2026_70364_MOESM4_ESM.zip › SourceFiles/Fig1G.tiff]

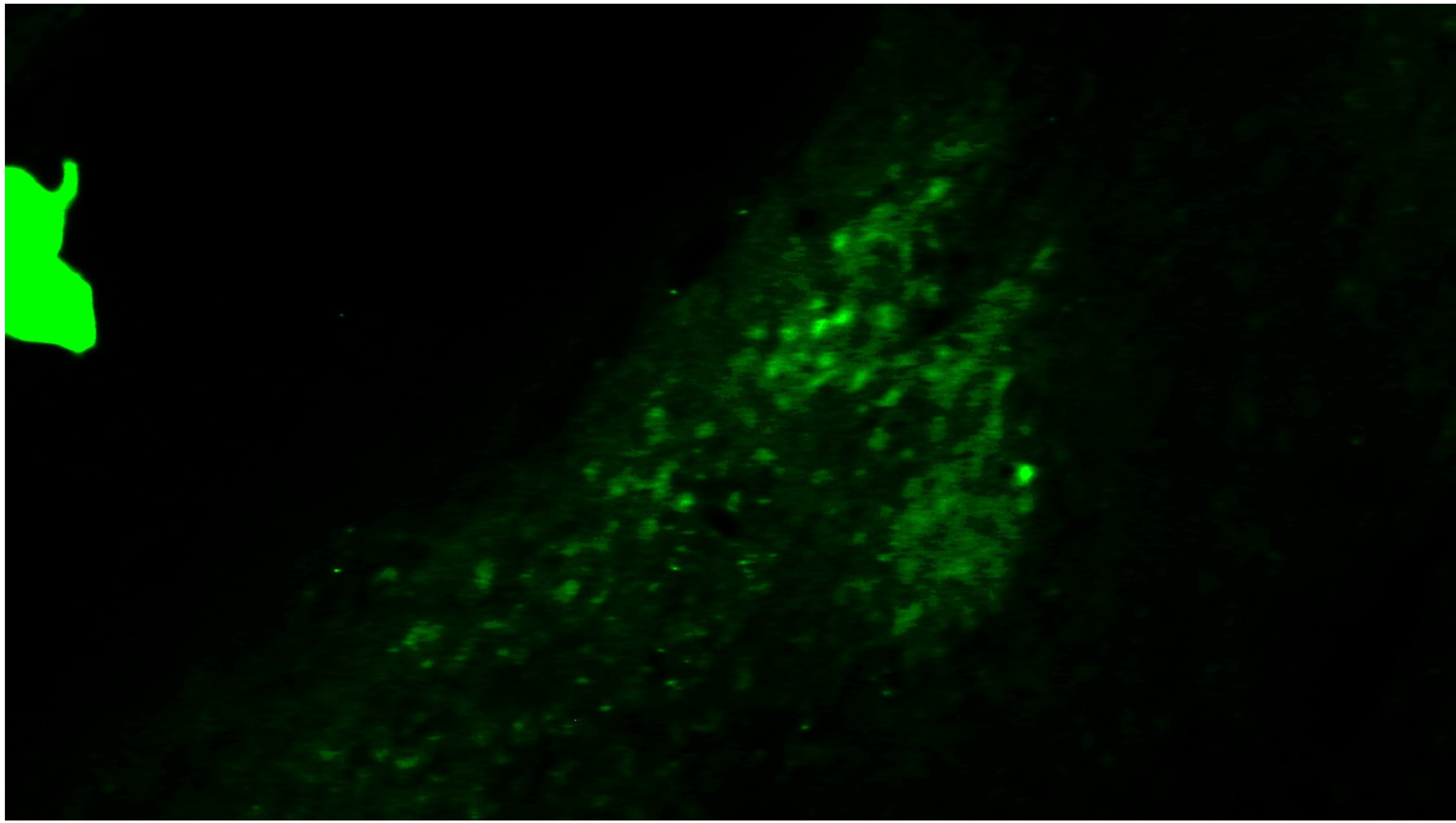

Supplement: Supplementary file 4 — Source data [file 41467_2026_70364_MOESM4_ESM.zip › SourceFiles/Fig 6F.tiff]

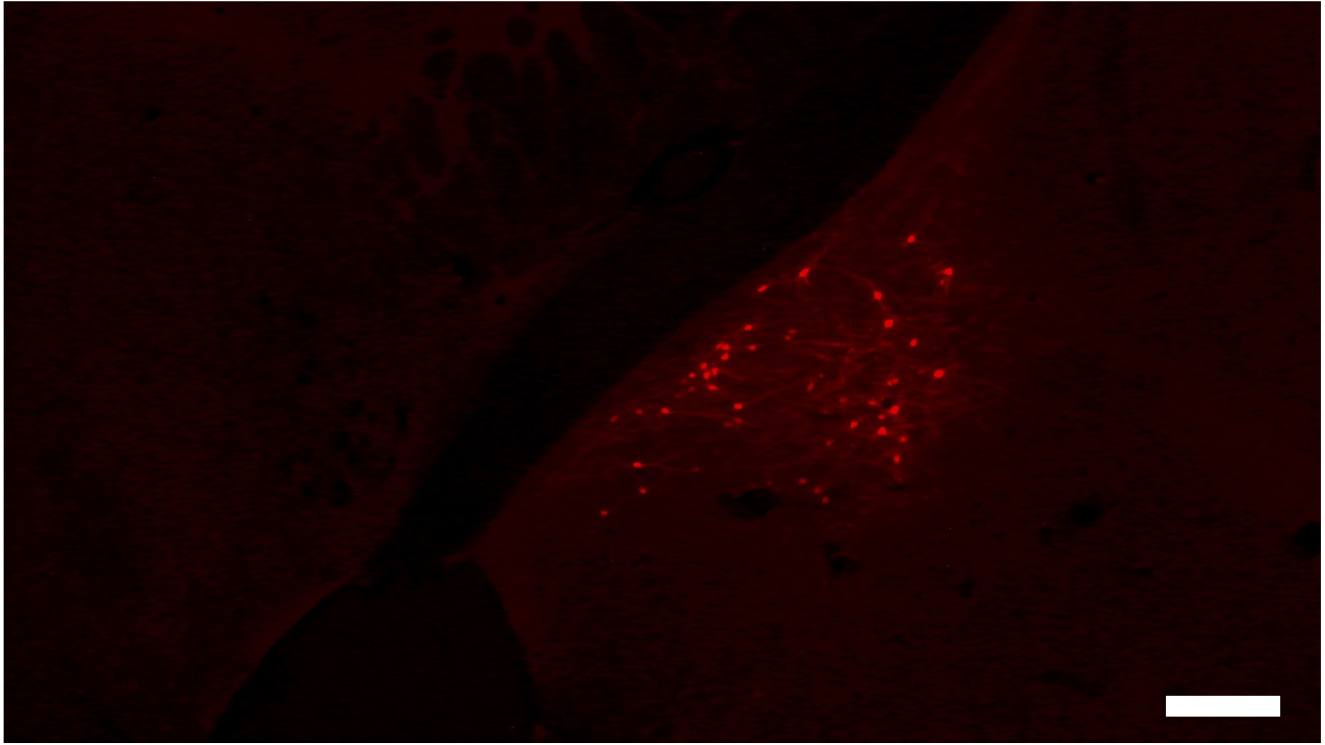

Supplement: Supplementary file 4 — Source data [file 41467_2026_70364_MOESM4_ESM.zip › SourceFiles/Fig5K.tiff]

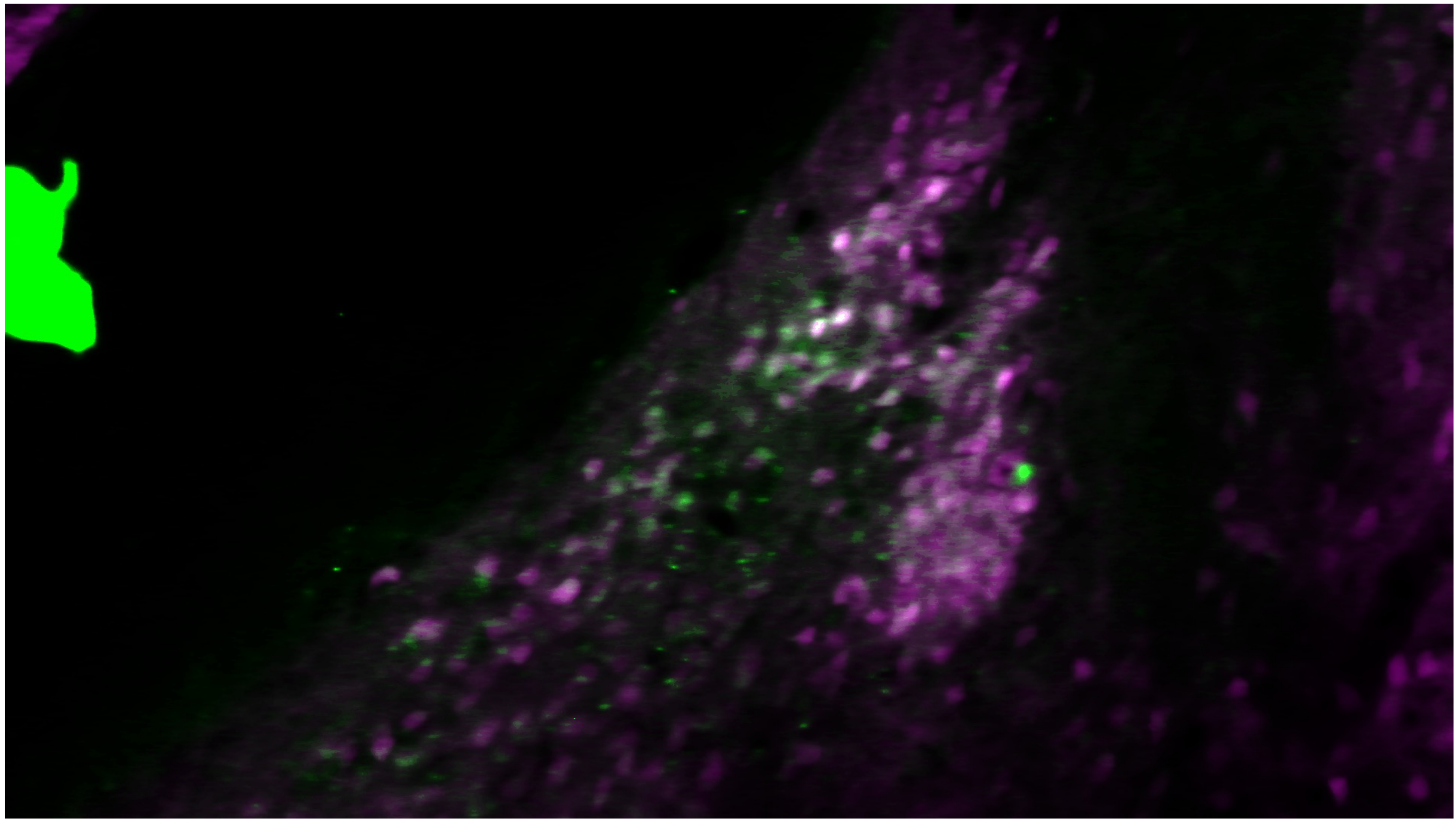

Supplement: Supplementary file 4 — Source data [file 41467_2026_70364_MOESM4_ESM.zip › SourceFiles/Fig 6G.tiff]

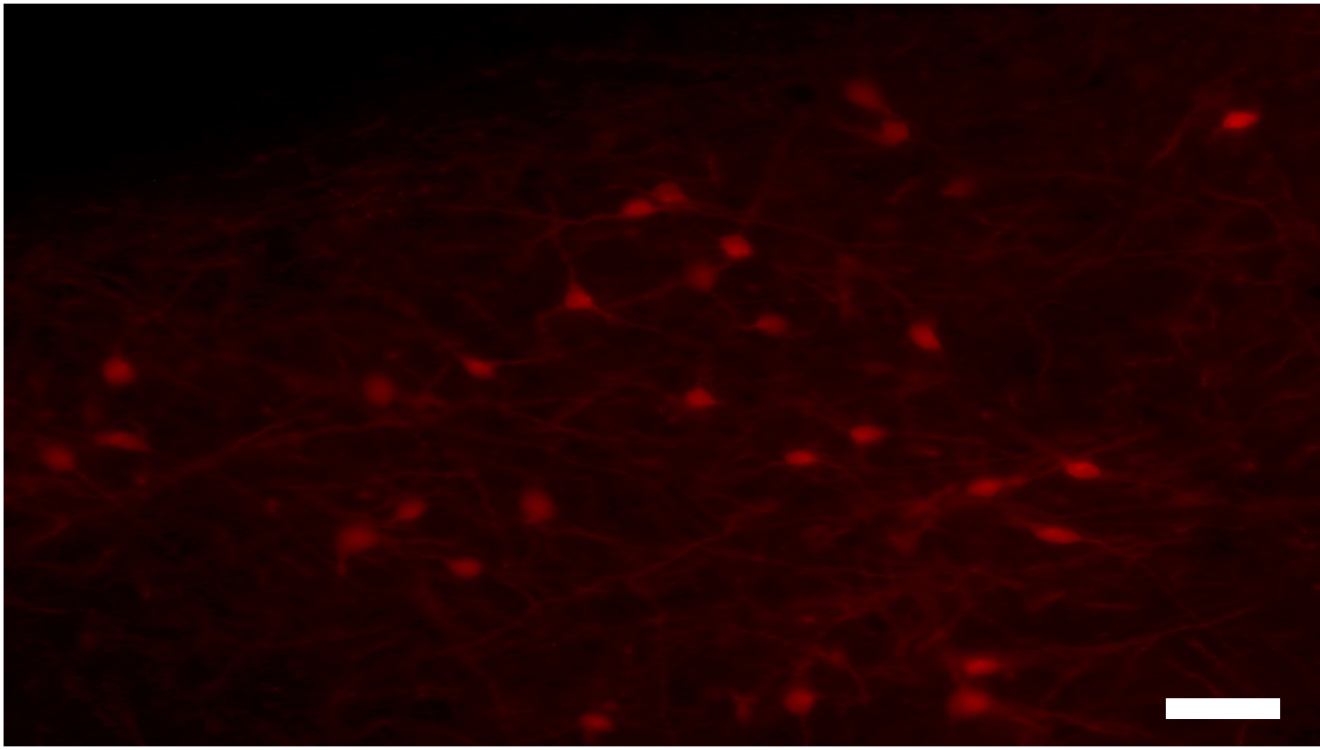

Supplement: Supplementary file 4 — Source data [file 41467_2026_70364_MOESM4_ESM.zip › SourceFiles/Fig5G.tiff]

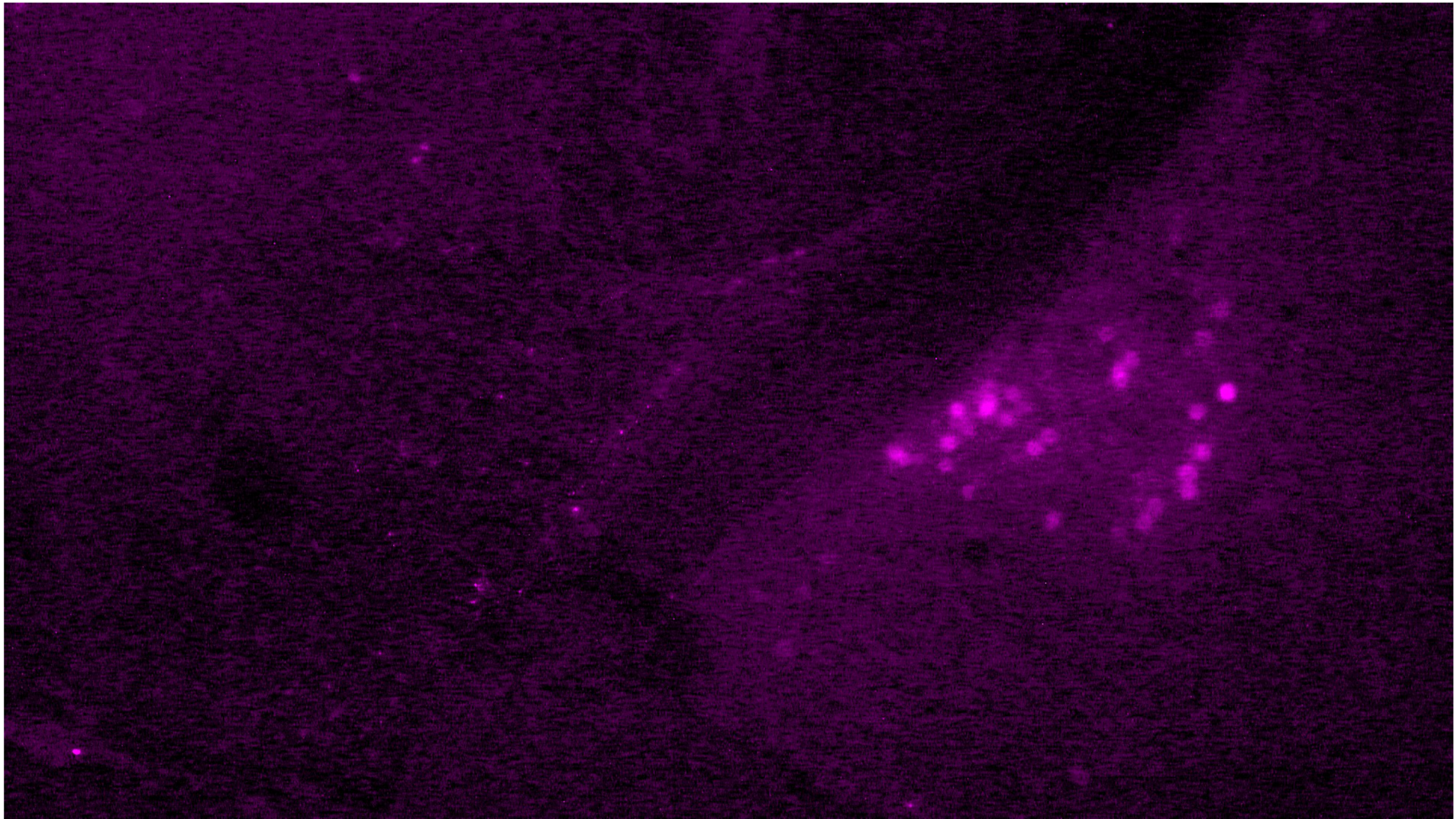

Supplement: Supplementary file 4 — Source data [file 41467_2026_70364_MOESM4_ESM.zip › SourceFiles/Fig 6K.tiff]

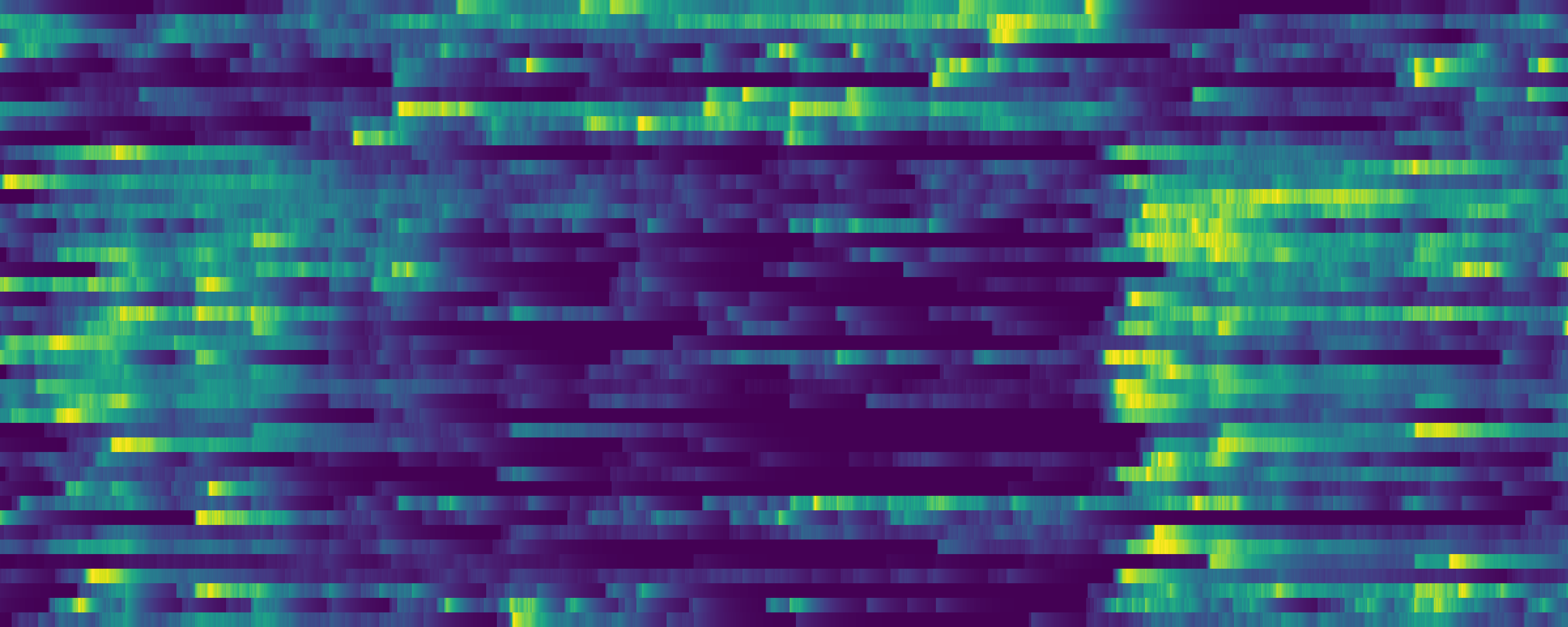

Supplement: Supplementary file 4 — Source data [file 41467_2026_70364_MOESM4_ESM.zip › SourceFiles/Figure2B.tiff]

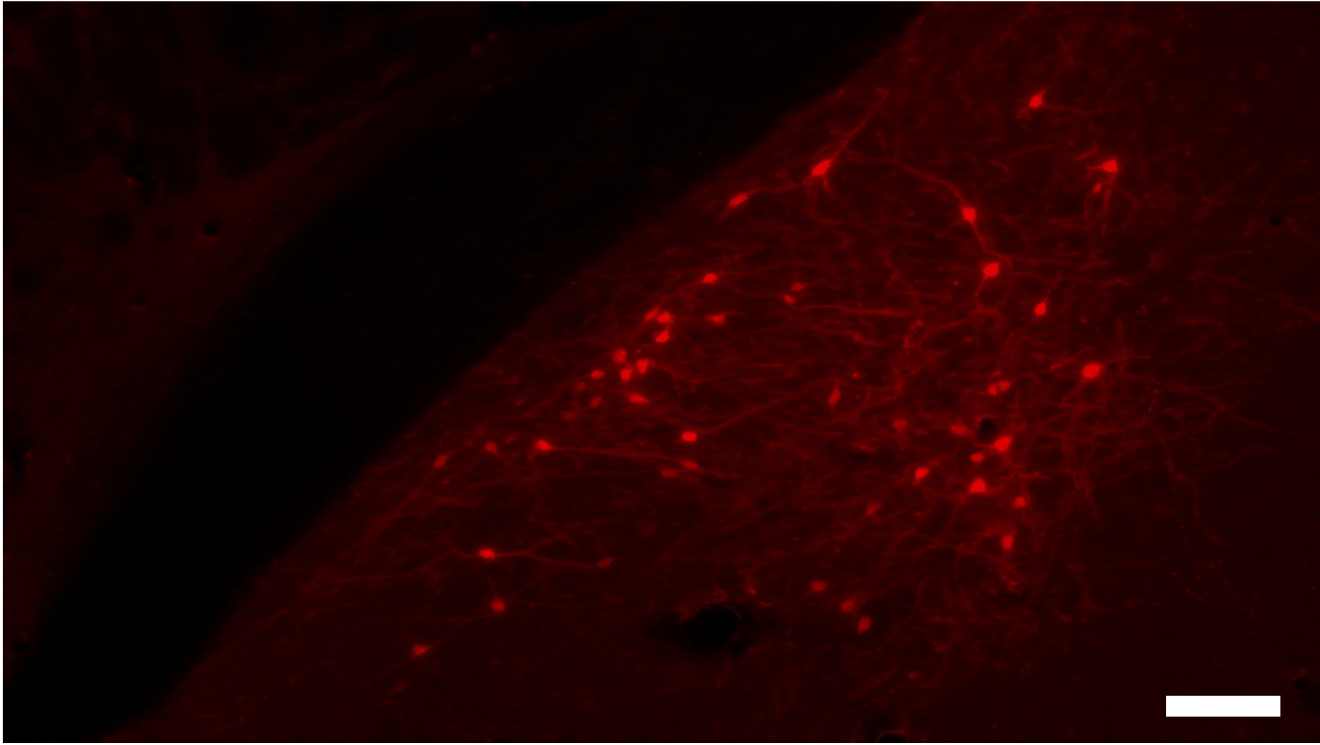

Supplement: Supplementary file 4 — Source data [file 41467_2026_70364_MOESM4_ESM.zip › SourceFiles/Fig5L.tiff]

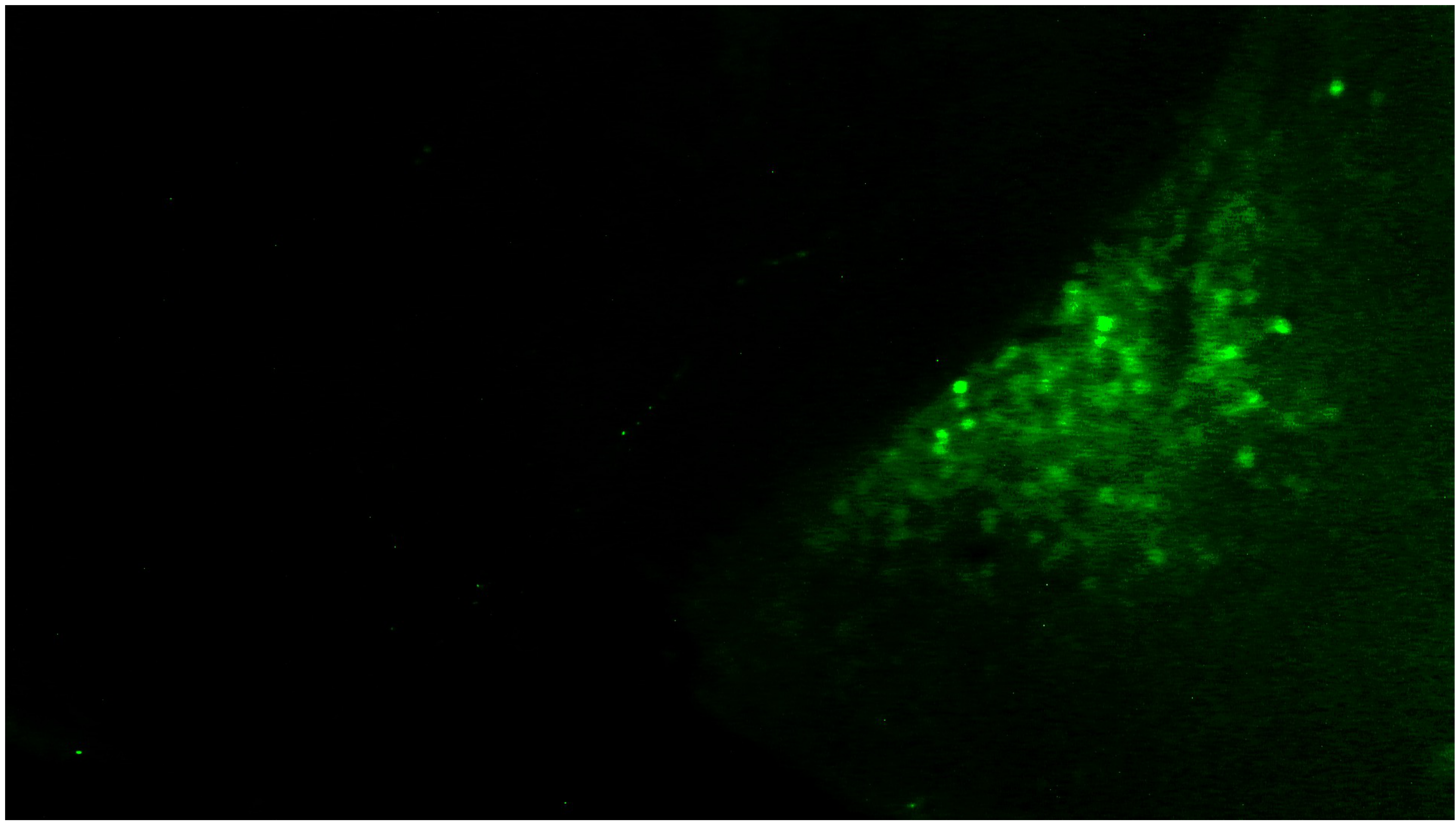

Supplement: Supplementary file 4 — Source data [file 41467_2026_70364_MOESM4_ESM.zip › SourceFiles/Fig 6L.tiff]

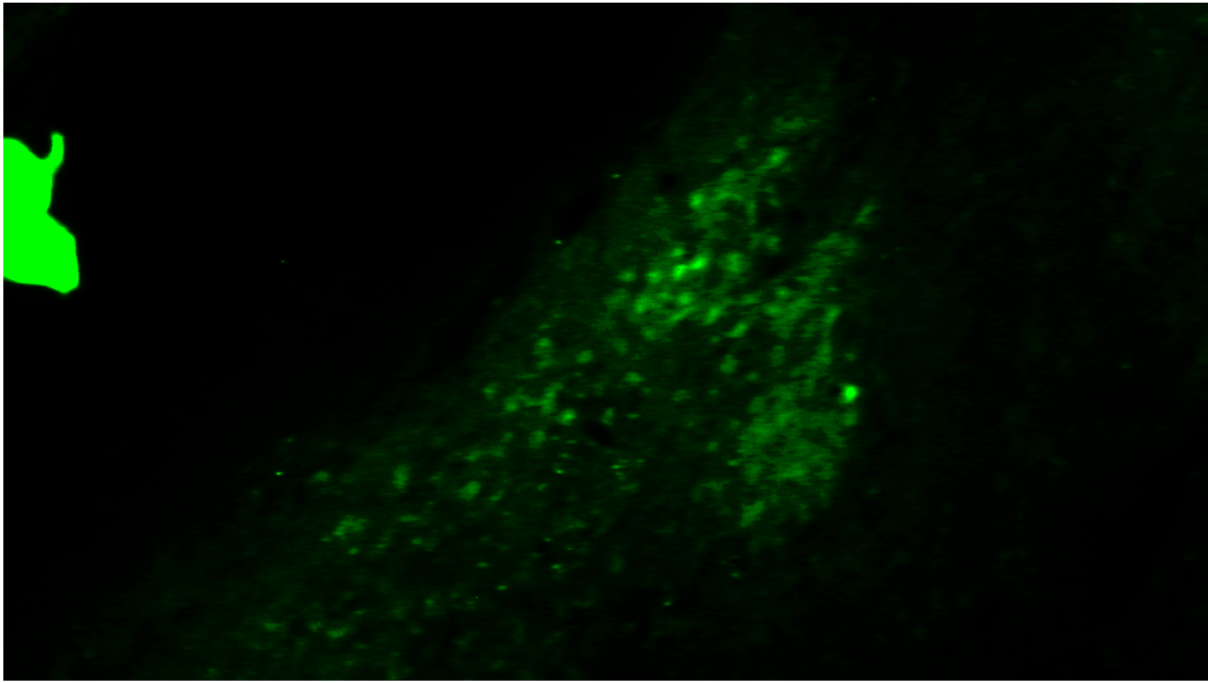

Supplement: Supplementary file 4 — Source data [file 41467_2026_70364_MOESM4_ESM.zip › SourceFiles/Fig 1F.tiff]

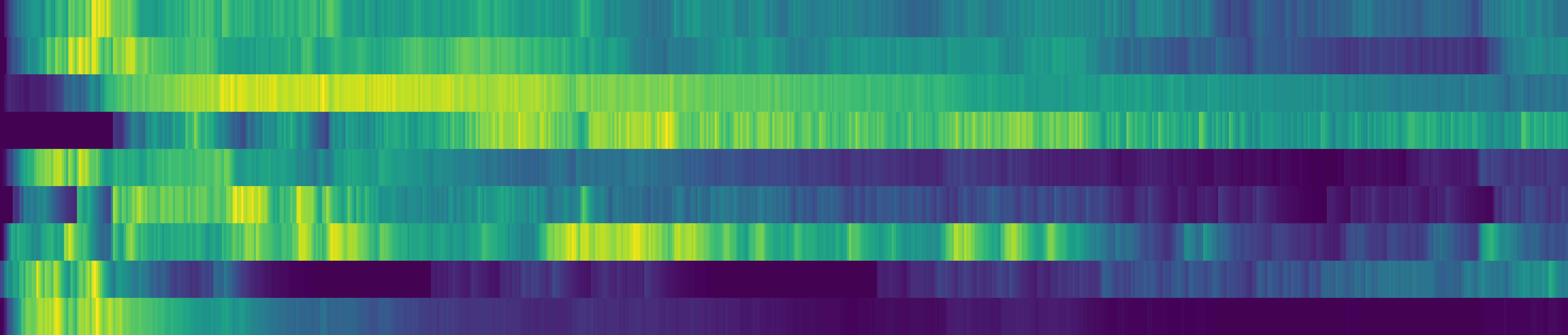

Supplement: Supplementary file 4 — Source data [file 41467_2026_70364_MOESM4_ESM.zip › SourceFiles/Figure3B.tiff]

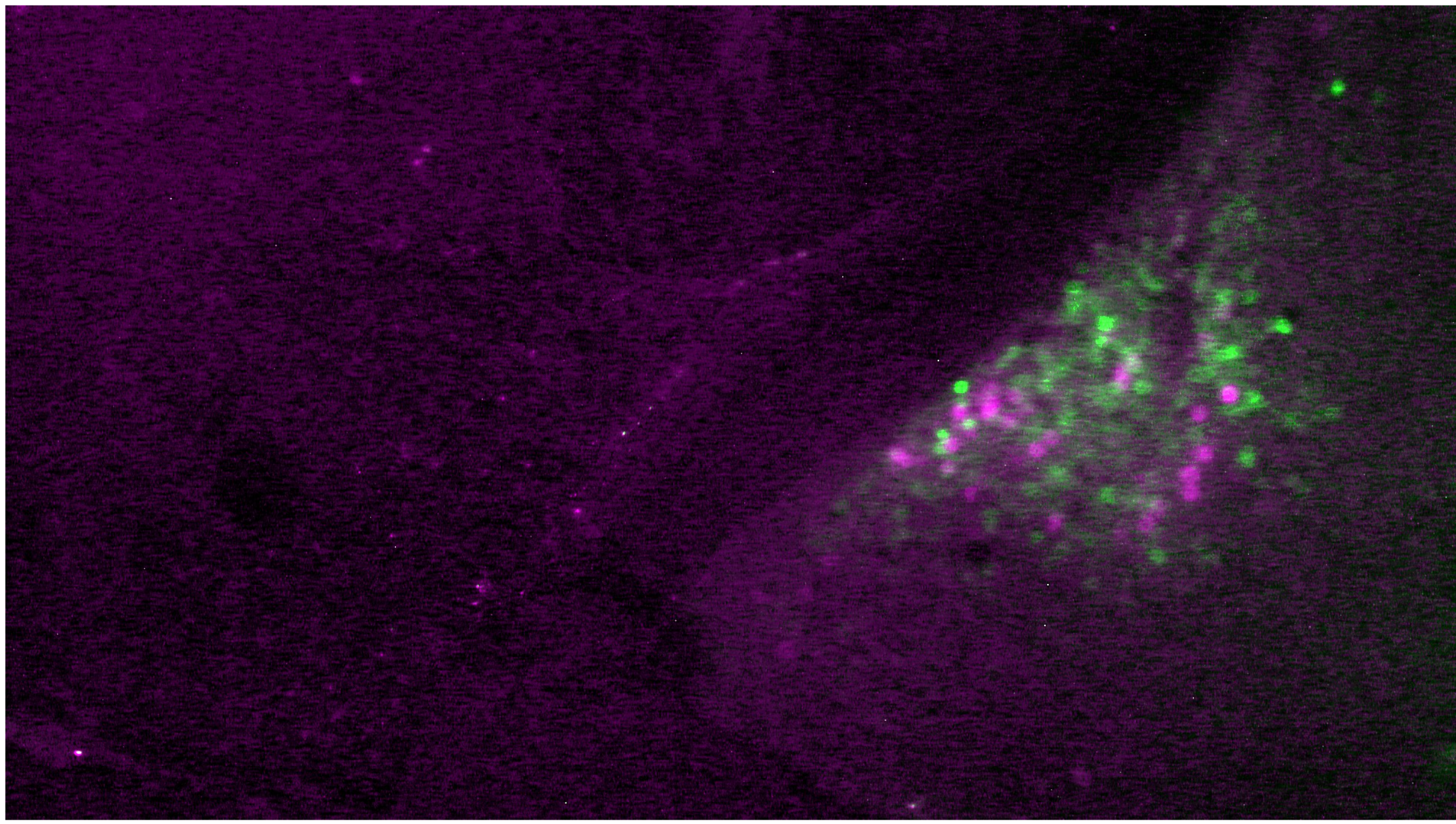

Supplement: Supplementary file 4 — Source data [file 41467_2026_70364_MOESM4_ESM.zip › SourceFiles/Fig 6M.tiff]

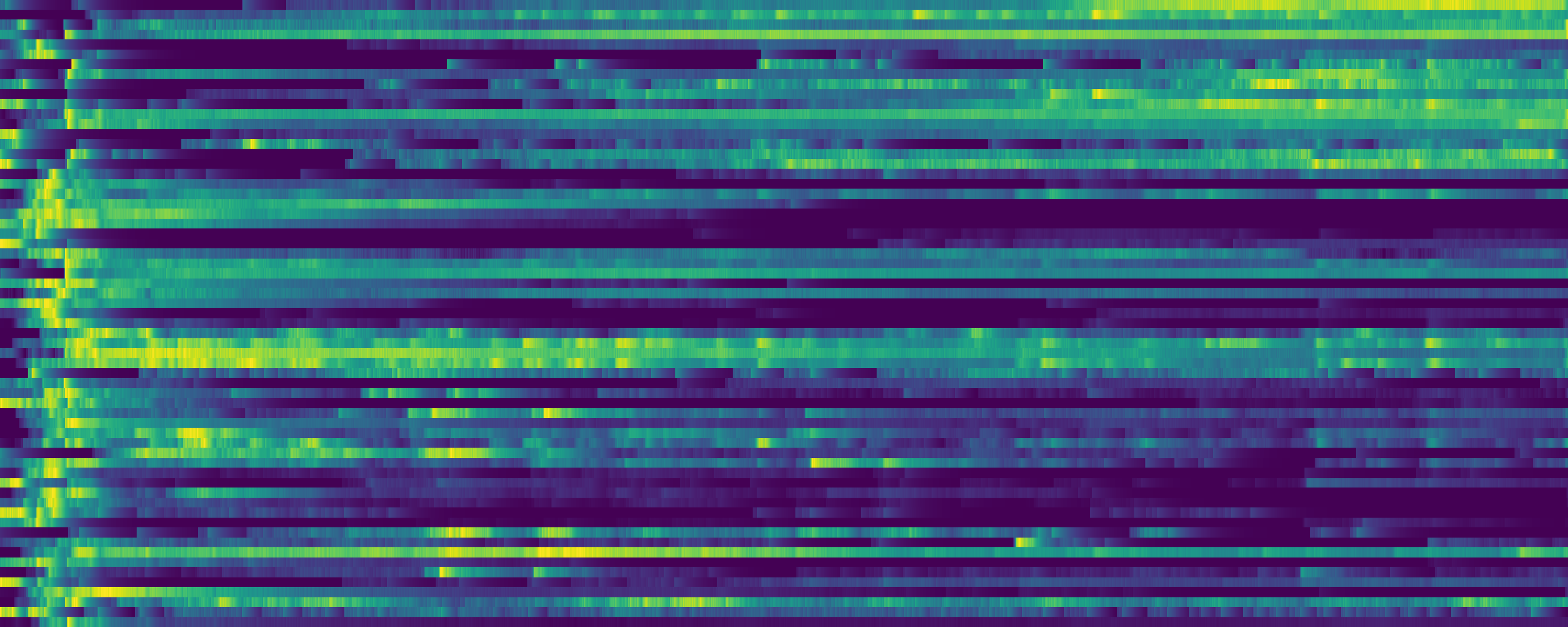

Supplement: Supplementary file 4 — Source data [file 41467_2026_70364_MOESM4_ESM.zip › SourceFiles/Figure2D.tiff]

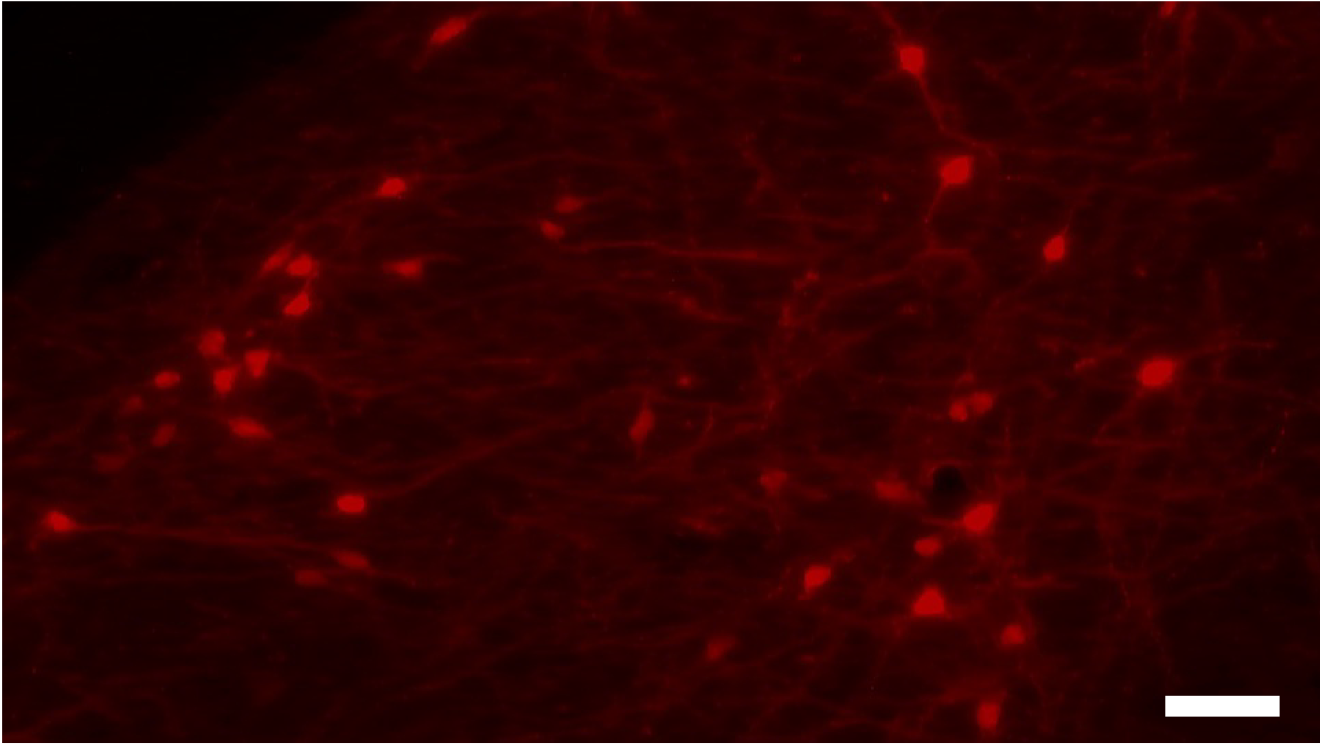

Supplement: Supplementary file 4 — Source data [file 41467_2026_70364_MOESM4_ESM.zip › SourceFiles/Fig5M.tiff]
