# Supplementary material for: The role of amygdala GABA neurons in controlling stress and reproduction in female mice
Source: Nat Commun. 2026 Mar 10;17:5690. doi: 10.1038/s41467-026-70364-9 (PMC13319269; doi:10.1038/s41467-026-70364-9)
Supplement: Supplementary file 3 — Transparent Peer Review file [file 41467_2026_70364_MOESM3_ESM.pdf]

nature portfolio

Peer Review File

The Role of Amygdala GABA Neurons in Controlling Stress and Reproduction in Female Mice

Corresponding Author: Professor Krasimira Tsaneva-Atanasova

This file contains all reviewer reports in order by version, followed by all author rebuttals in order by version.

Version 0:

Reviewer comments:

Reviewer #1

(Remarks to the Author)
Review of Yu et al., Amygdala GABA Neurons: Gatekeepers of Stress and Reproduction
The manuscript by Yu et al investigates the involvement of different populations of posterodorsal medial amygdala (MePD) neurons on luteinizing hormone (LH) pulse frequency. The authors use an impressive array of tools including in vivo Ca2+ imaging, optogenetics and modelling. The authors conclude that there are two anti-correlated GABAergic sub-populations in the MePD that dictate responses to both UCN3 neuron stimulation and restraint stress.
This study provides additional insight into how neural circuits in the MePD regulate LH pulse frequency and hence fertility. As such, this is important work. However, I have a few concerns, which are noted below.

Previous work from several of the authors from this group have suggested a different connectively pattern than that currently proposed (see Ivanova et al Endocrinology 2023). In that paper, the UNC3 neurons are proposed to be glutamatergic. However, in this paper, they are proposed to be GABAergic. Because of the complexity of the proposed circuit under study, it is not possible to delineate the connectively with in vivo optogenetics. As such, it is essential in my opinion that the authors conduct additional in vitro channelrhodopsin-assisted circuit mapping with patch clamp electrophysiology to conclusively show the true connectivity. In addition, these in vitro experiments would reveal 1) whether UNC3 neurons release GABA, glutamate or both and 2) the effect of endogenous UNC3 release on MePD GABA neuron excitability.

It wasn't until I read the supplementary Fig 8 that I appreciated how the miniscope imaging needed to be processed in order to exclude UNC3 expressing GABA neurons. While I am sure that this process does exclude a large number of these neurons, how can the authors be sure that all are excluded? In the UNC3 GABA neurons that remain, do these contribute to one of the clusters observed in Fig 2? At the very least, the data for these cells should be analysed and reported in Fig 2. Secondly, are the UNC3 cells excluded from the optogenetic stimulation data set also excluded from the stress data set? If they are, then also please show the response of the UNC3 neurons to stress.

I notice that the baseline activity of GABA neurons prior to optogenetic stimulation (Fig 1A) is much higher than the baseline activity of the neurons prior to stress. Why is that? It would be useful to determine if the response of these neurons in the optogenetic stimulation experiment can predict how these neurons respond to stress.

Many of the experiments in this manuscript rely on the targeting of specific neural populations with intersectional strategies. However, there is no quantification of the targeting of any of the viruses used (i.e. % coexpression). This quantification is required so that the reader can see how specific the virus expression is.

In addition to reporting effects on LH pulse frequency, it is also important to report how LH pulse height and mean LH levels are affected by the experimental manipulations.

(Remarks on code availability)

Reviewer #2

(Remarks to the Author)
The authors employ an elegant combination of in vivo optical methods, computational modelling, and hormone measurements to dissect the circuit mechanisms underlying the impact of stress on the MePD and downstream KNDy network. The study is timely and compelling, integrating multiple levels of analysis to provide insight into how stress modulates reproductive function. However, several key concerns related to specificity, controls, and interpretation must be addressed to ensure a robust and well-supported conclusion. I encourage the authors to consider these points carefully to strengthen their findings.

MAJOR POINTS
The authors should provide direct evidence confirming the specificity of their intersectional strategy. In Figure 1B-F, the fluorescence panels are labelled as UCN3 and VGAT, yet these signals merely represent red and green fluorescence, which may not necessarily correspond to the expected cell types. To support specificity, quantitative validation is needed, such as an overlap analysis of UCN3 immunohistochemical staining with red fluorescence to confirm accurate targeting.

The manuscript suggests that both optogenetic UCN3 stimulation and restraint stress induce similar functional connectivity changes and clustering patterns in MePD GABAergic populations. However, stress appears to drive a much stronger excitation of GABA neurons than UCN3 stimulation. Have the authors quantified the effects of stress and UCN3 stimulation on $\Delta F/F$ changes compared to baseline? A direct statistical comparison would clarify the relative strength of these manipulations. A more definitive test would be to silence UCN3 neurons during stress to assess whether the activation of GABAergic neurons and clustering patterns are abolished. While this may be beyond the scope of the current study, it should at least be discussed as a necessary future experiment.

Have the authors done sufficient controls to ensure that 1-photon light excitation of the calcium indicator does not also activate the opsin? Similarly, have they conducted control experiments to verify that their observed effects are due to activation of inhibitory neurons rather than simply light delivery? These controls are well-documented for Figures 5-6 but are missing for Figures 1-2.

Unless I am misunderstanding this, it seems that SLxCorr analysis allows for lag values spanning nearly the entire recording: $\tau \in \{-N+1,\ldots,-1,0,1,\ldots,N-1\}$. Given that neuronal interactions typically occur on much shorter timescales, could the authors justify this choice?
Have they tested whether restricting τ to a smaller, physiologically relevant window alters their clustering results? Additionally, to confirm that anticorrelated ensembles exist independently of the clustering approach, could the authors provide a standard correlation matrix (e.g., using Pearson correlation) computed over a biologically relevant lag window? This would help verify that clear anticorrelated subgroups are present in the raw data, strengthening confidence in the reported clustering results.

Given this, how can the authors be sure that a mutually inhibitory interaction in the model is a correct implementation? The anticorrelated activity may occur at a very long timescale that is not related to biologically plausible interactions between neurons. Further explanations and/or controls to justify this choice are needed.

The modelling approach assumes mutual inhibition between the two GABAergic populations, yet this is not independently validated, and given the long time lags in SLxCorr, the presence of direct monosynaptic inhibition is unclear. Is anticorrelation an emergent property of the model or an inevitable built-in feature given the model architecture (i.e., simply enforced by the mutual inhibition, which may be unwarranted)? Could the authors clarify how they validated the presence of mutual inhibition and whether alternative model architectures would result or not in anticorrelated activity?

The intersectional approaches to restrict expression to individual populations or mixed populations are elegant and strengthen the paper. However, more thorough controls are required to ensure the expression patterns in the expected neuron types are present. Fluorescence micrographs are often not sufficiently zoomed in or clear, and are not corroborated by additional controls (e.g., immunohistochemical staining to verify expression in the appropriate cell types).

The authors write: “We test these predictions using optogenetics and confirm that GABAergic neurons operate downstream of the UCN3 population, playing a crucial role in transmitting UCN3 signals to regulate luteinizing hormone (LH) pulse frequency.” However, I do not see an unequivocal experimental demonstration of UCN3 neurons acting upstream of GABA neurons. The paper would benefit from either stronger circuit mapping evidence or a fairer description of the findings.

The title could better reflect the actual findings of the paper.

MINOR POINTS
Instead of performing KS tests on individual mice and combining p-values with Fisher’s method for functional connectivity changes, a more straightforward approach would be to compare baseline vs. stress/stimulation using mice as samples (e.g., paired t-test or Wilcoxon signed-rank test). This would directly assess whether functional connectivity changes at the group level. Have the authors considered this approach?

The study uses hand-held restraint as a stressor, but this method can introduce variability due to experimenter handling

differences and potential habituation effects. Could the authors clarify the choice of this model (e.g., they used predator odour in Ivanova et al. 2021)?

The paper would benefit from a better introduction and discussion (with references) about previous work supporting the link between stress and infertility. Clarifying whether this happens with chronic or acute stress and how this relates to the current study (acute stress) would be useful.

Do the authors have any information on the behaviour of the mouse during calcium imaging baseline recordings? i.e., were they mostly still, moving, etc.?

Remove “opsin stimulation” in Figure 4 and replace it with “stimulation” and a different symbol. It is confusing given that this is a model.

Line 289: “Opposite” is probably a more appropriate word than “orthogonal.”

(Remarks on code availability)
I have not tested the code, but it includes a thorough README file with enough instructions for running. In principle, the code can be used to reproduce the figures related to the in silico model and the analysis of the calcium imaging data.

Reviewer #3

(Remarks to the Author)
I co-reviewed this manuscript with one of the reviewers who provided the listed reports. This is part of the Nature Communications initiative to facilitate training in peer review and to provide appropriate recognition for Early Career Researchers who co-review manuscripts.

(Remarks on code availability)

Reviewer #4

(Remarks to the Author)
In this manuscript, Yu et al. explore the role of UCN3 and GABAergic neurons in the medial amygdala posterodorsal subdivision (MePD) in regulating gonadotropin-releasing hormone (GnRH) pulse frequency, combining optogenetics, calcium imaging, and computational modeling to unravel how these neurons encode stress signals and modulate luteinizing hormone (LH) pulsatility. The study identifies complementary roles for two distinct GABAergic sub-populations and highlights the function between UCN3 and GABA signaling in the MePD. These findings provide new insights into the neural circuits underlying stress-induced reproductive suppression and have important scientific implications.

A key question is how the activation strength of UCN3 neurons regulates the mutual inhibition between the two distinct GABAergic subpopulations. This issue could be addressed experimentally.

Minor comments:
1.Fig. 1J should be clearly explained.
2.P5, line 105: no reference(s) is/are given for this statement, i.e. neurons response as a set of interconnected nodes (neurons) with connections can weighted by their functional interactions
3.P7, line 139:“Fig2D&E”# “Fig2E&F”
4.P7, line 119-120 pAAV-Syn- Flex-rc # pAAV-Syn- Flex - Chrimson - tdTomato?
5.P9, line 158: Please provide a brief introduction to the purpose and principles of the mathematical modeling framework for MePD circuitry.
6.Some paragraphs could be further streamlined to avoid lengthy descriptions. For example, P9 and P11, the description of the computational model could more clearly highlight its relationship to experimental data.
7.P17, line 312 AAV-EF1a-ChR2 # AAV-EF1a-DIO-ChR2
8.The fluorescent brain tissue images in Fig 5 E-F and K-M should display the optical fiber sites.
9.All optogenetic viruses used in the manuscript should provide evidence of their effectiveness.
10.Definitions or explanations of key terms (e.g., "GABAergic efferent neurons") could be included for non-specialist readers.
11.For critical experiments (e.g., optogenetic activation of GABAergic neurons leading to LH pulse suppression), increasing control group sample size could improve statistical power.
12.Some conclusions (e.g., identifying GABAergic neurons as downstream targets of UCN3 signaling) could be further discussed in the context of physiological and pathological conditions.
13.The classification of GABAergic neurons (e.g., efferent vs. interneurons) within the MePD could be discussed further for its broader applicability to other brain regions.
14.Additional discussion on the practical implications of these findings for reproductive health (e.g., stress-related infertility) could strengthen the manuscript.

(Remarks on code availability)

Version 1:

Reviewer comments:

Reviewer #1

(Remarks to the Author)
The revised manuscript by Yu and colleagues addresses several of my points raised in the first round of review. However, I still have some concerns which are noted below.

As noted in the first round of review, circuit mapping experiments are essential to define the connectivity between MePD neurons. I appreciate that this cannot be achieved for this current paper. However, if the arrangement MePD circuit cannot be confirmed by circuit mapping, this limits the conclusions which can be drawn. Parallel to this, I think that graphical representation of the MePD circuit shown in Figures 3 and 4 are misleading. While I appreciate these diagrams refer to the mathematical model, many readers will also assume they apply to the biological circuit. My concerns include the fact that UNC3 neurons co-release other fast neurotransmitters and that this is not shown in the diagrams/model or discussed in terms of its importance for circuit function. The fact that UNC3 optogenetic simulation effects will be partly mediated by these co-transmitters is also not discussed sufficiently.

Thank you for providing more details on how the miniscope imaging was analysed. It would also be informative if the authors could include the number of neurons analysed for each animal in each experiment, as well as the number of cells excluded from Ca^{2+} imaging analysis for each experiment.

I noticed in the revised manuscript (Figure 1J, Supp Fig 7, 8) that multiple trials of the same manipulation were performed in the same mouse. It appears that these are being treated as independent samples for statistical purposes. Is that correct? If so, it would be best to repeat the statistical analysis of these data with each animal contributing a single data point. Also, the repetition number for each animal varies. Animal G37 was stressed twice but not other animals? Animal G8 had 4 optogenetic experiments, but animal G7 only had 2. The rationale for this was not explained.

In the revised text, it is stated that “Heatmaps of calcium activity ($\Delta F/F0$) (left) in response to optogenetic stimulation of UCN3 neurons excluding GABA cells co-expressing UCN3 (see Supplementary Fig. 2) and (right) during restraint-induced stress.” I found this sentence hard to interpret. In the text, please add additional clarification that the optogenetic stimulation will target both UNC3 and UNC3+GABA co-expressing neurons. Please also provide addition clarification that the populations of neurons captured in the two imaging experiments (panel H vs Panel I) will be different.

The new data presented in the revised manuscript shows that “Varying the frequency of optogenetic stimulation of UCN3 neurons influenced the functional connectivity within the GABA network (Supplementary Fig. 7). Stimulation at 5 Hz produced no significant change compared with controls, 10 Hz resulted in a statistically significant increase, while 20 Hz reduced the effect, consistent with a depolarization block.”
The authors provide no evidence that 20Hz stimulation will induce a depolarisation block and I suggest that this statement is removed. Instead, this data calls into question whether certain neurotransmitters/neuropeptides are being released preferentially at these higher frequencies of stimulation.

Thankyou for reporting the additional LH data in supplementary Table 3. It would be great to also include the statistical comparisons for this data.

(Remarks on code availability)

Reviewer #2

(Remarks to the Author)
I thank the authors for this thorough revision, which has substantially improved the manuscript in terms of rigour, transparency, and clarity. I have only a few relatively minor comments remaining.

1) The similarity of the effects of stress and UCN3 stimulation on GABA neurons appears to be a central point of the study, and the causal involvement of GABAergic neurons in the stress cascade is suggested throughout the manuscript. However, I am not entirely convinced that this is discussed in a sufficiently balanced way, especially given the additional analyses and examples now included. The authors refer to a “comparable network reconfiguration”, and while the similarity in the anticorrelated clusters is indeed clear, the effects on overall activity (i.e. $\Delta F/F$) appear less consistent. For example, in Fig. 1A and 1K, stress induces a substantial increase in activity, whereas UCN3 stimulation produces very limited or virtually no increase. In any case, activity during stress seems 20-fold higher than UCN3 stimulation. In Fig. 2B, the increase following UCN3 stimulation seems more substantial. If the “rate” of GABA neurons is clearly less enhanced by UCN3 stimulation than

by stress, this discrepancy should be acknowledged and discussed, rather than assuming that the network state is entirely comparable. Potential explanations could include technical factors, or additional biological mechanisms such as neuromodulators released during stress but not during isolated UCN3 stimulation. As I mentioned in my earlier review, a direct way to assess causality would be to silence UCN3 neurons during stress to test whether GABAergic activation and the observed clustering patterns are abolished. While such an experiment is beyond the current scope, suggesting this approach in the Discussion could strengthen the manuscript.

2) 119. Given what you are comparing, shouldn't the sentence read: "Furthermore, stress induces a SIGNIFICANTLY HIGHER population time-averaged calcium activity than UCN3 stimulation"

3) The label "opsin stimulation" is still present in Fig.3. Please replace with "stimulation".

Overall, I believe the paper is close to acceptance, and a fairer treatment of point 3 above will further enhance its clarity, transparency and impact.

(Remarks on code availability)
I have not tested the code, but it appears well organised and clearly explained.

Reviewer #3

(Remarks to the Author)
I co-reviewed this manuscript with one of the reviewers who provided the listed reports. This is part of the Nature Communications initiative to facilitate training in peer review and to provide appropriate recognition for Early Career Researchers who co-review manuscripts.

(Remarks on code availability)

Reviewer #4

(Remarks to the Author)
The authors have convincingly addressed the revisions requested in my previous assessment. I have no further concerns.

(Remarks on code availability)

Version 2:

Reviewer comments:

Reviewer #1

(Remarks to the Author)
The authors have addressed my previous comments. I have no further concerns.

(Remarks on code availability)

Open Access This Peer Review File is licensed under a Creative Commons Attribution 4.0 International License, which permits use, sharing, adaptation, distribution and reproduction in any medium or format, as long as you give appropriate credit to the original author(s) and the source, provide a link to the Creative Commons license, and indicate if changes were made.
In cases where reviewers are anonymous, credit should be given to 'Anonymous Referee' and the source.
The images or other third party material in this Peer Review File are included in the article's Creative Commons license, unless indicated otherwise in a credit line to the material. If material is not included in the article's Creative Commons license and your intended use is not permitted by statutory regulation or exceeds the permitted use, you will need to obtain permission directly from the copyright holder.
To view a copy of this license, visit https://creativecommons.org/licenses/by/4.0/

Response to the reviewers

We would like to thank the reviewers for their constructive feedback. Below, we address each of their comments point by point

Reviewer #1

1. Previous work from several of the authors from this group have suggested a different connectivity pattern than that currently proposed (see Ivanova et al Endocrinology 2023). In that paper, the UNC3 neurons are proposed to be glutamatergic. However, in this paper, they are proposed to be GABAergic. Because of the complexity of the proposed circuit under study, it is not possible to delineate the connectively with in vivo optogenetics. As such, it is essential in my opinion that the authors conduct additional in vitro channelrhodopsin-assisted circuit mapping with patch clamp electrophysiology to conclusively show the true connectivity. In addition, these in vitro experiments would reveal 1) whether UNC3 neurons release GABA, glutamate or both and 2) the effect of endogenous UNC3 release on MePD GABA neuron excitability.

The challenging *in-vivo* study by Ivanova et al. 2023, utilised optogenetic activation of UCN3 neurons with intra-nuclear neuropharmacological antagonism of GABAergic or glutamatergic signalling. Both were affective at blocking the inhibitory effects of optical stimulation on UCN3 neurons on LH pulse frequency. The schematic circuitry referred to by the reviewer explained our novel neuropharmacological observations and the proposed potential glutamatergic nature of the UCN3 neurones was congruent with this neurochemical phenotype of UCN3 neurones in other brain regions, including the hypothalamic perifornical area. The cutting-edge GRIN-lens miniendoscopic technology used in the present study allows us to selectively visualise the actual activity of individual GABA neurones in the amygdala, the neurones of interest, and record their response to stress or optogenetic stimulation of the stress-related UCN3 neurones, placing us in a unique position to interrogate the circuitry. We examined the response of GABA neurones to optical stimulation of UCN3 neurons and found that some GABA neurons may co-express UCN3 (see response to 2nd comment) As the reviewer clearly recognises co-release of GABA *and* glutamate is prevalent (eg. Ceballos etal 2024; doi:10.1101/2024.03.27.587069) in the CNS. Naturally, we cannot rule out the possibility that UCN3 neurons co-express both neurotransmitters. Although in vitro electrophysiological patch-clamp experiments would indeed be valuable in elucidating the circuit, regrettably we are not in a position to perform them at this stage. We would therefore kindly ask the reviewer to consider our suggestion that such experiments may be beyond the scope of the present study, which is focused primarily on the activity of GABA neurons in the amygdala.

2. It wasn't until I read the supplementary Fig 8 that I appreciated how the miniscope imaging needed to be processed in order to exclude UNC3 expressing GABA neurons. While I am sure that this process does exclude a large number of these neurons, how can the authors be sure that all are excluded? In the UNC3 GABA neurons that remain, do these contribute to one of the clusters observed in Fig 2? At the very least, the data for these cells should be analysed and reported in Fig 2. Secondly, are the UNC3 cells excluded from the optogenetic stimulation data set also excluded from the stress data set? If they are, then also please show the response of the UNC3 neurons to stress.

To ensure the exclusion of as many UNC-3 neurons expressing GABA as possible from our analysis, we applied two independent filtering approaches. The first filter involved calculating the power spectral density of individual neurons and excluding those that showed significant power around the frequency of our stimulation protocol (5-second on/off intervals; 0.1 Hz). The second filter used unsupervised clustering, which identified a distinct population of neurons exhibiting clear oscillatory activity in phase with the stimulation. Neurons identified by either filter were excluded from all subsequent analyses. This filtering pipeline is now clearly described in the Methods section. For added clarity and conciseness, we also provide an illustrative figure showing the activity of these neurons in the Supplementary Information (Supplementary Fig. 2), which is referenced in the caption of Figure 1 and in line 130-131.

Regarding the reviewer's second comment, UCN3-expressing GABAergic neurons cannot be directly identified in recordings from acutely stressed animals. These neurons may therefore contribute to the observed clusters. Nonetheless, the presence of two distinct clusters aligned with the stimulation protocol suggests that the functional role of UCN3 GABAergic neurons during acute stress is limited. This interpretation is further supported by our functional intersectional study (Fig. 5H–J).

3. I notice that the baseline activity of GABA neurons prior to optogenetic stimulation (Fig 1A) is much higher than the baseline activity of the neurons prior to stress. Why is that? It would be useful to determine if the response of these neurons in the optogenetic stimulation experiment can predict how these neurons respond to stress.

In Figure 1, we present scaled $\Delta F/F_0$ traces normalized to the 0–1 range using the minimum and maximum values from the entire duration of the experiment. While this approach allows for visual comparison between baseline and intervention periods, it renders comparisons across different experiments invalid. In this example, the response to stress greatly exceeds baseline activity, making the baseline activity appear dwarfed after normalization. In contrast, during UCN3 stimulation, activity during the intervention looks more comparable to baseline levels. We have quantified the effects of stress and UCN3 stimulation on $\Delta F/F_0$ changes compared to baseline and our results are now presented in Fig 1K.

4. Many of the experiments in this manuscript rely on the targeting of specific neural populations with intersectional strategies. However, there is no quantification of the targeting of any of the viruses used (i.e. % coexpression). This quantification is required so that the reader can see how specific the virus expression is. In addition to reporting effects on LH pulse frequency, it is also important to report how LH pulse height and mean LH levels are affected by the experimental manipulations.

We have now included the quantification of the % co-expression of the viruses used in the revised manuscript. Lines 94-98, 278-283 and 305-310. See Supplemental Fig. 1, 11 and 12 for the supportive histology. We have also added the data relating to LH pulse amplitude and mean LH levels. See Supplementary Tables 3 and 4.

Reviewer #2

1. The authors should provide direct evidence confirming the specificity of their intersectional strategy. In Figure 1B-F, the fluorescence panels are labelled as UCN3 and VGAT, yet these signals merely represent red and green fluorescence, which may not necessarily correspond to the expected cell types. To support specificity, quantitative validation is needed, such as an overlap analysis of UCN3 immunohistochemical staining with red fluorescence to confirm accurate targeting.

In the revised manuscript we have provided the overlapping analysis of the UCN3 immunohistochemical staining with the red fluorescence to confirm accurate targeting. See Supplementary Fig. 1. The appropriate methodology is outlined in lines 677-698.

2. The manuscript suggests that both optogenetic UCN3 stimulation and restraint stress induce similar functional connectivity changes and clustering patterns in MePD GABAergic populations. However, stress appears to drive a much stronger excitation of GABA neurons than UCN3 stimulation. Have the authors quantified the effects of stress and UCN3 stimulation on ΔF/F changes compared to baseline? A direct statistical comparison would clarify the relative strength of these manipulations. A more definitive test would be to silence UCN3 neurons during stress to assess whether the activation of GABAergic neurons and clustering patterns are abolished. While this may be beyond the scope of the current study, it should at least be discussed as a necessary future experiment.

We thank the reviewer for their constructive comment. Following up on this comment, we calculated the change of the time-averaged $\Delta F/F_0$ relative to baseline under conditions of stress and UCN3 stimulation. Our results indicate a significant difference between the two manipulations at short timescales (<90 seconds), which diminishes by 120 seconds. Results of the analysis can be found in Fig. 1K.

3. Have the authors done sufficient controls to ensure that 1-photon light excitation of the calcium indicator does not also activate the opsin? Similarly, have they conducted control experiments to verify that their observed effects are due to activation of inhibitory neurons rather than simply light delivery? These controls are well-documented for Figures 5-6 but are missing for Figures 1-2.

The Inscopix system we use was designed to circumvent or minimise the type of cross-activation the reviewer refers to, providing of course that one uses the appropriate AAV constructs. Indeed, note that one-photon GECI excitation is typically achieved with light powers that are approximately an order of magnitude lower than opsin excitation, which allows for simultaneous use of GCaMP6 and Chrimson with minimal cross-talk. We have ensured that our experimental design minimises the risk of cross-activation, and we can confirm the following:

1. **Wavelength Separation: As mentioned, the excitation light for GCaMP6s (~480 nm) and Chrimson-tdTomato (~590 nm) are well-separated, ensuring minimaloverlap in their activation spectra (Siciliano and Tye 2018, https://doi.org/10.1016/j.alcohol.2018.05.013). The excitation light for GCaMP6s does not activate Chrimson-tdTomato, and we have used nVoke Optics, which optimises this separation further, with the 455 ± 8 nm excitation for GCaMP6s and 620 ± 30 nm for Chrimson. This setup is also supported by previous studies (Jimenez et al. 2020, https://www.nature.com/articles/s41467-020-17270-w; Stamatakis et al. 2018, https://doi.org/10.3389/fnins.2018.00496; Vidal-Ortiz et al. 2024, https://doi.org/10.1093/sleep/zsad217)**
2. **Optogenetic activation of inhibitory neurons is not purely due to light delivery. Figures 5-6 already include light-only control experiments where no response is observed with the control virus, showing that light delivery itself does not cause neuronal responses. Therefore, the light delivery controls in Figures 5-6 serve as evidence that light alone is not driving the effects observed in Figures 1-2, ie., GABA neuronal activity that correlates with LH pulse dynamics.**
3. **Irradiance Differences: The irradiance for the GCaMP6s imaging system (0-2 mW) is much lower than that for Chrimson-tdTomato, further reducing the possibility of cross-activation during imaging. This significant difference in irradiance between the two light sources ensures that GCaMP6s excitation does not inadvertently activate Chrimson-tdTomato.**
4. **Additionally, one can see from Figure 1 A&J, that the behaviour of the GABAergic neurons significantly changes from the baseline period and subsequent optical stimulation.**
5. **With respect to the control experiments to verify that our observed effects are due to activation of the inhibitory neurons rather than simply light delivery, we had carried out those experiments using the appropriate control viruses and they are now included in figure 1J.**

4. Unless I am misunderstanding this, it seems that SLxCorr analysis allows for lag values spanning nearly the entire recording: $\tau \in \{-N+1,...,-1,0,1,...,N-1\}$. Given that neuronal interactions typically occur on much shorter timescales, could the authors justify this choice? Have they tested whether restricting τ to a smaller, physiologically relevant window alters their clustering results? Additionally, to confirm that anticorrelated ensembles exist independently of the clustering approach, could the authors provide a standard correlation matrix (e.g., using Pearson correlation) computed over a biologically relevant lag window? This would help verify that clear anticorrelated subgroups are present in the raw data, strengthening confidence in the reported clustering results

We thank the reviewer for their comment. Using Pearson correlation (zero-lag) to assess functional connectivity reveals two anticorrelated clusters, consistent with the results obtained using SLxCorr. This confirms the presence of clearly anticorrelated groups within the GABAergic population. To simplify the presentation of our results (see also comment 5), we have revised our analysis pipeline to use Pearson correlation matrices for assessing the network's functional connectivity and identifying functional clusters, while retaining the SLxCorr analysis in the Supplementary Information. Our results and conclusions remain unchanged.

5. Given this, how can the authors be sure that a mutually inhibitory interaction in the model is a correct implementation? The anticorrelated activity may occur at a very long timescale that is not related to biologically plausible interactions between neurons. Further explanations and/or controls to justify this choice are needed.
The modelling approach assumes mutual inhibition between the two GABAergic populations, yet this is not independently validated, and given the long time lags in SLxCorr, the presence of direct monosynaptic inhibition is unclear. Is anticorrelation an emergent property of the model or an inevitable built-in feature given the model architecture (i.e., simply enforced by the mutual inhibition, which may be unwarranted)? Could the authors clarify how they validated the presence of mutual inhibition and whether alternative model architectures would result or not in anticorrelated activity?

As we discussed in the point above, our analysis results reveal two anticorrelated neuronal clusters regardless of whether we use lagged cross-correlation or Pearson correlation (zero-lag). Given the GABAergic identity of these neurons, mutual inhibitory interactions between the two clusters are expected and represent a justifiable explanation for the anticorrelated activity. We used our model to investigate whether unidirectional inhibition might give rise to anticorrelated activity, but our results indicate that this mechanism alone is insufficient.

6. The intersectional approaches to restrict expression to individual populations or mixed populations are elegant and strengthen the paper. However, more thorough controls are required to ensure the expression patterns in the expected neuron types are present. Fluorescence micrographs are often not sufficiently zoomed in or clear, and are not corroborated by additional controls (e.g., immunohistochemical staining to verify expression in the appropriate cell types).

Please see response to point 1 above. The new figure includes immunohistochemical staining and has higher magnification images.

7. The authors write: "We test these predictions using optogenetics and confirm that GABAergic neurons operate downstream of the UCN3 population, playing a crucial role in transmitting UCN3 signals to regulate luteinizing hormone (LH) pulse frequency." However, I do not see an unequivocal experimental demonstration of UCN3 neurons acting upstream of GABA neurons. The paper would benefit from either stronger circuit mapping evidence or a fairer description of the findings.

We appreciate the reviewer's comments and have revised the manuscript to reflect a fairer description of the aims and findings. See abstract and lines 480-489 (discussion).

8. The title could better reflect the actual findings of the paper.

We have changed the title of the manuscript in accordance with the reviewer's recommendation, thank you.

<u>Minor comments</u>:
9. Instead of performing KS tests on individual mice and combining p-values with Fisher's method for functional connectivity changes, a more straightforward approach would be to compare baseline vs. stress/stimulation using mice as samples (e.g., paired t-test or Wilcoxon signed-rank test). This would directly assess whether functional connectivity changes at the group level. Have the authors considered this approach?

We thank the reviewer for their suggestion. Following the revision of our pipeline to quantify functional connectivity using Pearson correlation, we have also updated our statistical analysis to compare baseline and intervention periods across control, UCN3-stimulated, and stressed animals. The results are presented in Figure 1, and the methodology is detailed in the Methods section.

11. The study uses hand-held restraint as a stressor, but this method can introduce variability due to experimenter handling differences and potential habituation effects. Could the authors clarify the choice of this model (e.g., they used predator odour in Ivanova et al. 2021)?

We appreciate the concerns of the reviewer. However, we know from our experience that hand-held restraint does decrease hypothalamic arcuate multi-unit activity volleys, an electrophysiological correlate of GnRH pulse generator activity and LH pulsatility in mice. All the *in-vivo* mini-endoscopic experiments were carried out by one individual which will have minimised experimenter handling differences, and we know from our considerable experience that mice do not readily habituate to this type of restraint in comparison to normal gentle handling associated with attaching and detaching the mini-endoscope camera. Ideally, we would have liked to have used predator odour, but the size of the Inscopix system is not compatible with a fume cupboard that is essential for this stressor as used by Ivanova et al 2021.

12. The paper would benefit from a better introduction and discussion (with references) about previous work supporting the link between stress and infertility. Clarifying whether this happens with chronic or acute stress and how this relates to the current study (acute stress) would be useful.

In line with the reviewer's recommendation, we have revised the manuscript accordingly. See lines, 41-44 and 494-498.

13. Do the authors have any information on the behaviour of the mouse during calcium imaging baseline recordings? i.e., were they mostly still, moving, etc.?

The mice are extensively habituated to the Inscopix system, so the vast majority of the time they are still. We have never observed any difference in baseline neuronal activity, whether the animal is still, moving or indeed sleeping.

14. Remove “opsin stimulation” in Figure 4 and replace it with “stimulation” and a different symbol. It is confusing given that this is a model.

We have revised Figures 3 and 4 and replaced the term “opsin stimulation” with “stimulation” and used a different symbol to minimise confusion.

15. Line 289: “Opposite” is probably a more appropriate word than “orthogonal.”

We have reworded the text to avoid using the term ‘orthogonal’. The revised sentence now reads: “our results suggest that although UCN3 and GABAergic neuronal populations overlap, they serve distinct functional roles within the MePD circuit, with both being critical for relaying inhibitory signals to the hypothalamic GnRH pulse generator. While independent activation of either population inhibits the GnRH pulse generator, activation of UCN3 neurons that co-express GABA is insufficient to elicit this effect.”. See lines 319-323.

Reviewer #4

1. A key question is how the activation strength of UCN3 neurons regulates the mutual inhibition between the two distinct GABAergic subpopulations. This issue could be addressed experimentally.

We thank the reviewer for their constructive suggestion. We used varying frequencies of optogenetic stimulation of UCN3 neurons to examine how activation strength influences GABA neuron activity. Our data show that stimulation at 5, 10, or 20 Hz does not significantly alter the correlation between the average activity of the two GABA clusters (Supplementary Fig. 8). However, stimulation frequency does affect overall functional connectivity within the GABA network (Supplementary Fig. 7). Specifically, 5 Hz stimulation produces no significant change relative to control animals, while 10 Hz stimulation leads to a measurable alteration in functional connectivity. Further increasing the frequency to 20 Hz reduces the effect on the functional connectivity, consistent with a depolarization block effect.

<u>Minor comments</u>:

2. Fig. 1J should be clearly explained.

Our revised statistical analysis is now clearly explained in the caption and methodology section.

3. P5, line 105: no reference(s) is/are given for this statement, i.e. neurons response as a set of interconnected nodes (neurons) with connections can weighted by their functional interactions

We referenced a recent review paper. R. Yuste, R Cossart, and E Yaksi. "Neuronal ensembles: Building blocks of neural circuits." ***Neuron*** **112.6 (2024): 860-861.**

4. P7, line 139:“Fig2D&E”# “Fig2E&F”

Correction made

5. P7, line 119-120 pAAV-Syn- Flex-rc # pAAV-Syn- Flex - Chrimson - tdTomato?

Correction made

6. P9, line 158: Please provide a brief introduction to the purpose and principles of the mathematical modeling framework for MePD circuitry.

We updated the first paragraph of the section “Mutually inhibitory interactions between GABA populations drive anti-correlated activity” to clarify the purpose of the mathematical model. We emphasise that the model is used to explore the mechanisms underlying the observed data. We also reference our previous work, in which we introduce the MePD model and provide further technical details.

7. Some paragraphs could be further streamlined to avoid lengthy descriptions. For example, P9 and P11, the description of the computational model could more clearly highlight its relationship to experimental data.

We would like to thank the reviewer for their helpful suggestion. We have updated P9 to provide a more precise introduction to the model and clarify the purpose of our in-silico investigations. Also, on P11, we explicitly emphasize that KNDy activity is a reliable proxy for LH pulsatility, reinforcing how our model is firmly grounded in and supported by experimental evidence.

8. P17, line 312 AAV-EF1a-ChR2 # AAV-EF1a-DIO-ChR2

Correction made

9. The fluorescent brain tissue images in Fig 5 E-F and K-M should display the optical fiber sites.

Correction made, see revised figures.

10. All optogenetic viruses used in the manuscript should provide evidence of their effectiveness.

We have now included the effectiveness of the optogenetic viruses as % co-expression of the viruses used in the revised manuscript in lines 94-98, 278-283 and 305-310 and Supplementary Fig. 1, 11, 12 for the supportive histology.

11. Definitions or explanations of key terms (e.g., "GABAergic efferent neurons") could be included for non-specialist readers.

We refer to the neurons that project their axons outside the MePD circuit to transmit the signal to other regions of the brain, such as hypothalamic KNDy, as GABA efferent neurons. For clarity we have described these as efferent projection neurons in the revised manuscript. See revised lines 161-162 and 175-178.

12. For critical experiments (e.g., optogenetic activation of GABAergic neurons leading to LH pulse suppression), increasing control group sample size could improve statistical power.

As suggested by the reviewer, additional experiments have been conducted to improve statistical power. See revised Figures 5 and 6.

13. Some conclusions (e.g., identifying GABAergic neurons as downstream targets of UCN3 signaling) could be further discussed in the context of physiological and pathological conditions.

We appreciate the reviewer's recommendation and have added the appropriate text. See lines 480-484.

14. The classification of GABAergic neurons (e.g., efferent vs. interneurons) within the MePD could be discussed further for its broader applicability to other brain regions.

We discuss the diversity of GABA neurons within the MePD and the relevance of our findings and modelling work to other brain regions (see lines 445–450).

15. Additional discussion on the practical implications of these findings for reproductive health (e.g., stress-related infertility) could strengthen the manuscript.

We appreciate the reviewer's recommendation and have added the appropriate text. See lines 494-498.

Responses to reviewers' comments

We would like to thank the reviewers for their further comments on our manuscript. We have carefully considered all the feedback and have provided point-by-point responses below in bold text. Where appropriate, we have revised the manuscript accordingly to address the reviewers' suggestions.

Reviewer 1 (Remarks to the Author):

The revised manuscript by Yu and colleagues addresses several of my points raised in the first round of review. However, I still have some concerns which are noted below.

As noted in the first round of review, circuit mapping experiments are essential to define the connectivity between MePD neurons. I appreciate that this cannot be achieved for this current paper. However, if the arrangement MePD circuit cannot be confirmed by circuit mapping, this limits the conclusions which can be drawn. Parallel to this, I think that graphical representation of the MePD circuit shown in Figures 3 and 4 are misleading. While I appreciate these diagrams refer to the mathematical model, many readers will also assume they apply to the biological circuit. My concerns include the fact that UNC3 neurons co-release other fast neurotransmitters and that this is not shown in the diagrams/model or discussed in terms of its importance for circuit function. The fact that UNC3 optogenetic simulation effects will be partly mediated by these co-transmitters is also not discussed sufficiently.

> **Response: We thank the reviewer for the useful comments. We agree that, in the absence of circuit-mapping data, the precise arrangement of MePD connections cannot be conclusively established, and we have taken steps to ensure that our modeling framework is not misinterpreted as a direct anatomical claim. We have revised the captions for Figures 3 and 4 to state explicitly that these diagrams represent the *in-silico* model architecture rather than experimentally validated connectivity.**
>
> **Additionally, to clarify how input of the UCN3 neurons is modeled we describe biological interpretation of the input function in the methods section. The description includes a fast excitatory component, representing potential co-release of fast neurotransmitters, and a slow component reflecting neuropeptide release (lines 816-818). We note it in results as well (lines 196-198). We have incorporated text in the Discussion addressing the potential role of fast excitatory neurotransmitters co-release for the MePD circuit function (lines 457-467). To**

further avoid the confusion between the mathematical abstraction and the biological circuit, we now include an additional figure (Figure 7) that illustrates a plausible MePD circuit organisation based on current experimental evidence. We also added text in the discussion that addresses potential co-release of GABA neurotransmitters that is prompted by our statistical analysis (lines 505-507).

Thank you for providing more details on how the miniscope imaging was analysed. It would also be informative if the authors could include the number of neurons analysed for each animal in each experiment, as well as the number of cells excluded from Ca2+ imaging analysis for each experiment.

Response: We thank the reviewer for this helpful suggestion. We have now included Supplementary Table 1 and 2, that summarize the number of neurons identified for each animal and each trial for stimulation and restraint, respectively. For stimulation condition we also provide the number of neurons that were excluded from our analysis based on our filtering criteria for co-expression of UCN3 and GABA (Supplementary Table 1).

I noticed in the revised manuscript (Figure 1J, Supp Fig 7, 8) that multiple trials of the same manipulation were performed in the same mouse. It appears that these are being treated as independent samples for statistical purposes. Is that correct? If so, it would be best to repeat the statistical analysis of these data with each animal contributing a single data point. Also, the repetition number for each animal varies. Animal G37 was stressed twice but not other animals? Animal G8 had 4 optogenetic experiments, but animal G7 only had 2. The rationale for this was not explained.

Response: We thank the reviewer for these thoughtful comments. We have now revised the analysis using a mixed-effects model, which accounts for the fact that multiple trials were performed on the same animal (lines 787-793), and Figure 1 has been updated accordingly. Due to the complexity of the experimental setup, it was not always possible to obtain the same number of experimental trials from each animal. Our aim was to conduct at least two independent stimulation trials per animal, with a maximum of four trials, we indicate it in the text (lines 663-665).

For the stress protocol, we performed a single trial per animal to minimize unnecessary distress, in line with the 3Rs principle. However, in the case of animal G37, the restraint stress was applied twice to assess whether this limited repetition would result in any habituation. As no habituation was observed, we

decided to include the repeated trial in our analysis. These details have now been included in the Methods section (lines 673-675).

In the revised text, it is stated that "Heatmaps of calcium activity (ΔF/F0) (left) in response to optogenetic stimulation of UCN3 neurons excluding GABA cells co-expressing UCN3 (see Supplementary Fig. 2) and (right) during restraint-induced stress."
I found this sentence hard to interpret. In the text, please add additional clarification that the optogenetic stimulation will target both UNC3 and UNC3+GABA co-expressing neurons. Please also provide addition clarification that the populations of neurons captured in the two imaging experiments (panel H vs Panel I) will be different.

Response: The sentence has now been revised for clarity and to explicitly state that the neuronal populations in the two cases are distinct, with the number of neurons involved in each case noted (lines 134-138).

The new data presented in the revised manuscript shows that "Varying the frequency of optogenetic stimulation of UCN3 neurons influenced the functional connectivity within the GABA network (Supplementary Fig. 7). Stimulation at 5 Hz produced no significant change compared with controls, 10 Hz resulted in a statistically significant increase, while 20 Hz reduced the effect, consistent with a depolarization block." The authors provide no evidence that 20Hz stimulation will induce a depolarisation block and I suggest that this statement is removed. Instead, this data calls into question whether certain neurotransmitters/neuropeptides are being released preferentially at these higher frequencies of stimulation.

Response: We thank the reviewer for their helpful comment. The text has been modified to address these issues (lines 161-164). Additionally, we added discussion regarding the involvement of potential neurotransmitters/neuropeptides (457-467, 505-508).

Thank you for reporting the additional LH data in supplementary Table 3. It would be great to also include the statistical comparisons for this data.

Response: We thank the reviewer for their suggestion. Supplementary Tables 5 and 6 have been updated to indicate statistically significant differences.

Reviewer 2 (Remarks to the Author):

I thank the authors for this thorough revision, which has substantially improved the manuscript in terms of rigour, transparency, and clarity. I have only a few relatively minor comments remaining.

1) The similarity of the effects of stress and UCN3 stimulation on GABA neurons appears to be a central point of the study, and the causal involvement of GABAergic neurons in the stress cascade is suggested throughout the manuscript. However, I am not entirely convinced that this is discussed in a sufficiently balanced way, especially given the additional analyses and examples now included. The authors refer to a “comparable network reconfiguration”, and while the similarity in the anticorrelated clusters is indeed clear, the effects on overall activity (i.e. ΔF/F) appear less consistent. For example, in Fig. 1A and 1K, stress induces a substantial increase in activity, whereas UCN3 stimulation produces very limited or virtually no increase. In any case, activity during stress seems 20-fold higher than UCN3 stimulation. In Fig. 2B, the increase following UCN3 stimulation seems more substantial. If the “rate” of GABA neurons is clearly less enhanced by UCN3 stimulation than by stress, this discrepancy should be acknowledged and discussed, rather than assuming that the network state is entirely comparable. Potential explanations could include technical factors, or additional biological mechanisms such as neuromodulators released during stress but not during isolated UCN3 stimulation. As I mentioned in my earlier review, a direct way to assess causality would be to silence UCN3 neurons during stress to test whether GABAergic activation and the observed clustering patterns are abolished. While such an experiment is beyond the current scope, suggesting this approach in the Discussion could strengthen the manuscript.

Response: We thank the reviewer for this constructive comment. We have now expanded the Discussion to acknowledge that the overall magnitude of GABAergic activity ($\Delta F/F_0$) differs between stress and UCN3 stimulation, despite the comparable network reconfiguration observed (lines 423-428).

Furthermore, we have added your suggestion that future studies could directly test the causal role of UCN3 neurons by silencing them during stress exposure (lines 483-485).

2) 119. Given what you are comparing, shouldn’t the sentence read: “Furthermore, stress induces a SIGNIFICANTLY HIGHER population time-averaged calcium activity than UCN3 stimulation”

Response: Text has been revised accordingly (lines 124-125).

3) The label “opsin stimulation” is still present in Fig.3. Please replace with “stimulation”.

Response: We thank the reviewer for pointing it out, the figure 3 has been revised accordingly.

Overall, I believe the paper is close to acceptance, and a fairer treatment of point 3 above will further enhance its clarity, transparency and impact.

Reviewer #2 (Remarks on code availability):

I have not tested the code, but it appears well organised and clearly explained.

Response: Thank you for this positive comment. We appreciate the reviewer's assessment that the code is well organised and clearly explained.

Reviewer #3 (Remarks to the Author):

I co-reviewed this manuscript with one of the reviewers who provided the listed reports. This is part of the Nature Communications initiative to facilitate training in peer review and to provide appropriate recognition for Early Career Researchers who co-review manuscripts.

Response: Thank you very much.

Reviewer #4 (Remarks to the Author):

The authors have convincingly addressed the revisions requested in my previous assessment. I have no further concerns.

Response: Thank you for this positive appraisal of our manuscript.
